# Enhanced dependency of KRAS‐mutant colorectal cancer cells on RAD51‐dependent homologous recombination repair identified from genetic interactions in *Saccharomyces cerevisiae*

**DOI:** 10.1002/1878-0261.12040

**Published:** 2017-03-27

**Authors:** Murugan Kalimutho, Amanda L. Bain, Bipasha Mukherjee, Purba Nag, Devathri M. Nanayakkara, Sarah K. Harten, Janelle L. Harris, Goutham N. Subramanian, Debottam Sinha, Senji Shirasawa, Sriganesh Srihari, Sandeep Burma, Kum Kum Khanna

**Affiliations:** ^1^ Signal Transduction Laboratory QIMR Berghofer Medical Research Institute Brisbane Australia; ^2^ School of Natural Sciences Griffith University Nathan Australia; ^3^ Division of Molecular Radiation Biology Department of Radiation Oncology University of Texas Southwestern Medical Center Dallas TX USA; ^4^ Department of Cell Biology Faculty of Medicine Fukuoka University Japan; ^5^ Institute for Molecular Bioscience The University of Queensland St. Lucia Australia

**Keywords:** colorectal cancer, DNA damage response, homologous recombination repair, *KRAS*, *RAD51*, therapeutic vulnerability

## Abstract

Activating *KRAS* mutations drive colorectal cancer tumorigenesis and influence response to anti‐EGFR‐targeted therapy. Despite recent advances in understanding Ras signaling biology and the revolution in therapies for melanoma using BRAF inhibitors, no targeted agents have been effective in *KRAS*‐mutant cancers, mainly due to activation of compensatory pathways. Here, by leveraging the largest synthetic lethal genetic interactome in yeast, we identify that *KRAS‐*mutated colorectal cancer cells have augmented homologous recombination repair (HRR) signaling. We found that KRAS mutation resulted in slowing and stalling of the replication fork and accumulation of DNA damage. Moreover, we found that KRAS‐mutant HCT116 cells have an increase in MYC‐mediated RAD51 expression with a corresponding increase in RAD51 recruitment to irradiation‐induced DNA double‐strand breaks (DSBs) compared to genetically complemented isogenic cells. *MYC* depletion using RNA interference significantly reduced IR‐induced RAD51 foci formation and HRR. On the contrary, overexpression of either HA‐tagged wild‐type (WT) MYC or phospho‐mutant S62A increased RAD51 protein levels and hence IR‐induced RAD51 foci. Likewise, depletion of RAD51 selectively induced apoptosis in HCT116‐mutant cells by increasing DSBs. Pharmacological inhibition targeting HRR signaling combined with PARP inhibition selectivity killed *KRAS*‐mutant cells. Interestingly, these differences were not seen in a second isogenic pair of *KRAS* WT and mutant cells (DLD‐1), likely due to their nondependency on the *KRAS* mutation for survival. Our data thus highlight a possible mechanism by which *KRAS‐*mutant‐dependent cells drive HRR *in vitro* by upregulating MYC‐RAD51 expression. These data may offer a promising therapeutic vulnerability in colorectal cancer cells harboring otherwise nondruggable *KRAS* mutations, which warrants further investigation *in vivo*.

**Abbreviations**
Alt‐NHEJ: alternative NHEJ
DDR: DNA damage response
DSBs: DNA double‐strand breaks
HRR: homologous recombination repair
MT: mutant
NHEJ: nonhomologous end joining
PI: propidium iodide
WT: wild‐type

## 1 Introduction

Activating Ras mutations are major contributors in cellular transformation and among the most frequently mutated drivers across multiple tumors including colorectal cancer (Schubbert *et al*., 2007). Ras proteins are small GTPases that shuttle between the plasma membrane and cytoplasm (McCormick, 1993a,b), depending on external and internal stimuli, to activate various effector proteins resulting in multiple cellular signaling responses including growth, proliferation, survival, differentiation, and morphogenesis (Bos *et al*., 2007; Cherfils and Zeghouf, 2013). The Ras superfamily members, *HRAS*,* KRAS*, and *NRAS*, are frequently mutated in codons 12 and 13 in exon 1 and less frequently in codon 61 which enable perturbation of the intrinsic GTPase activity of Ras proteins resulting in reductions in GTP hydrolysis capacity, and hence a constitutively active protein (Scheffzek *et al*., 1997).

Approximately 40–45% of colorectal cancers harbor *KRAS* mutations (De Roock *et al*., 2011), and mutation status is predictive of resistance to anti‐EGFR‐based targeted therapy in patients with metastatic colorectal cancer receiving cetuximab or panitumumab (Misale *et al*., 2014). Because of its high prevalence in human tumors, targeting mutant Ras itself has stimulated an intense research effort over the years, but to date, mutant Ras has not been a druggable target. Alternately, agents that block downstream effectors of Ras proteins through their canonical mitogen‐activated protein kinases (MAPKs) such as BRAF and MEK1/2 as well as phosphoinositide 3 kinases (PI3K) such as PI3K, AKT, or mTOR inhibitors have rapidly entered clinical trials (Knickelbein and Zhang, 2015). The efficacy of these inhibitors has been poor due to the activation of alternative oncogenic Ras‐mediated pathways, suggesting that inhibition of multiple downstream targets is required. Despite this, in recent years through genomic profiling of Ras‐mutant tumors and RNAi‐mediated gene silencing, many investigators have highlighted Ras‐dependent signaling in mutant tumors which could be targeted for therapeutic intervention by taking advantage of the synthetic lethality concept, whereby simultaneous loss of two genes results in cell death, but deletion of either individually does not impact cell viability. As such, TBK1 (Barbie *et al*., 2009), STK33 (Scholl *et al*., 2009), PLK1 (Luo *et al*., 2009), GATA2 (Kumar *et al*., 2012), TAK1 (Singh *et al*., 2012), CDK4 (Puyol *et al*., 2010), BCL‐XL (Corcoran *et al*., 2013), STAT3‐cMET (Van Schaeybroeck *et al*., 2014) as well as APC proteosome complex (Luo *et al*., 2009) could be viable targets for clinical development in *KRAS*‐mutant tumors.

The yeast *Saccharomyces cerevisiae* is a lower eukaryotic model with a simple compact genome, providing a powerful genetic system to understand the functional biology of thousands of genes via genetic deletion studies (Ooi *et al*., 2006). This yeast system has been used to discover synthetic lethal interactions among multiple genes to identify genetic interactions that can recapitulate similar genetic functions in mammalian systems. Similar to mammalian Ras proteins, yeast *S. cerevisiae* contains two Ras proteins, Ras1 and Ras2, which play a central role in controlling cAMP activity (Toda *et al*., 1985), and through MAPK signaling, both proteins regulate diploid pseudohyphal growth and haploid invasive growth, respectively (Gimeno *et al*., 1992; Stanhill *et al*., 1999). These studies and others in yeast have contributed enormously to our understanding of RAS signaling in humans. The similarity between yeast and human Ras proteins supports utilization of the yeast model system to study Ras genetic interactions in humans.

As direct pharmacological inhibition of the onco‐Ras proteins has thus far been unsuccessful in clinical trials, by leveraging the largest yeast synthetic lethal interactome (Costanzo *et al*., 2010; Deshpande *et al*., 2013), we explored genetic interactions in yeast to identify putative candidates for Ras‐interacting genes in mammalian cells. We report that the DNA double‐strand break repair (DSB repair) by homologous recombination (HR) pathway was enhanced in *KRAS*‐mutant colorectal cancer cells through hyperactivation of c‐MYC. Targeting RAD51, a downstream effector of homologous recombination repair (HRR) or CHK1 inhibition, thus may offer a novel strategy in killing mutant *KRAS‐*dependent colorectal cancers. Because the links between RAD51 and mutant *KRAS*‐mediated signaling was not known in the context of DNA damage response (DDR) regulation, these results also provide the first evidence on the role of *KRAS* mutations in hyperactivating HRR.

## 2 Materials and methods

### 2.1 Reagents

AZD6244, BEZ235, RI‐1, and AZD2281 were purchased from Selleck Chemicals LLC (Houston, TX, USA). siRNAs were purchased from Shanghai Gene Pharma (Shanghai, China). Lipofectamine^®^ RNAiMAX and Lipofectamine^®^ LTX with Plus™ Reagents were purchased from Life Technologies, Carlsbad (CA, USA) and CellTiter 96^®^ AQueous One Solution Cell Proliferation Assay from Promega Corporation, Fitchburg (WI, USA). The HA‐c‐MYC WT and S62A expression constructs were a gift from Professor Wuhan Xiao, Institute of Hydrobiology, Chinese Academy of Sciences.

### 2.2 Antibodies

The following antibodies were used in this study: RAD51 (GTX70230; GeneTex, Inc., Irvine, CA, USA), EMD Millipore, Billerica, MA, USA: RAD51 (PC130), γH2AX S139 (05‐636); Cell Signaling Technology, Inc., Danvers, MA, USA antibodies: PARP (#9542), pAKT S473 (#4060), AKT(#9272), pERK1/2 (#4370), ERK1/2 (#4695), HA (#3724), pP53 S15 (#9284), pCHK1 S345 (#2348), cleaved caspase 3 (#9664); Bethyl Laboratories, Inc., Montgomery, TX, USA antibodies: pKAP1 S824 (A300‐767A‐T), pRPA32 S4/S8 (A300‐245A), pRPA32 S33 (A300‐246A); and others: Cox‐IV (PN926‐42214, LI‐COR Biosciences, Lincoln, NE, USA), C‐MYC (AB32072), anti‐BrdU (ab6326; Abcam, Melbourne, Vic. Australia), 53BP1 (NB100‐304; Novus Biologicals, Littleton, CO, USA ) and anti‐BrdU (347580; Becton, Dickinson and Company, Franklin Lakes, NJ, USA).

### 2.3 Sequence alignment

Clustal Omega (http://www.ebi.ac.uk/Tools/msa/clustalo/) was used to align all the sequences.

### 2.4 Cell culture

The isogenic colorectal cancer cell lines, HCT116, HKh‐2, HKe‐3 DLD‐1, and DKs‐8, were obtained from Professor Senji Shirasawa (Fukuoka University, Japan) under a material transfer agreement and maintained in DMEM supplemented with 10% FBS. Other colorectal cancer lines were obtained from Professor Barbara Leggett (QIMR Berghofer, Australia). All cell lines were regularly tested for mycoplasma infection and authenticated using short tandem repeat profiling by scientific services at QIMR Berghofer Medical Research Institute.

### 2.5 Reverse transcriptase quantitative PCR

RNA was extracted using RNeasy Mini Kit (Qiagen, Venlo, Limburg, the Netherlands), and cDNA was synthesized using the SuperScript III First‐Strand Synthesis System (Life Technologies) according to the manufacturer's instructions. RT‐qPCR was performed on a LightCycler 480 (Roche, Basel, Switzerland) using SYBR Green (Roche) and normalized to β‐actin as an internal control (Table S1).

### 2.6 Ingenuity pathway analysis

Ingenuity pathway analysis was performed using the Ingenuity Pathway Analysis^®^ (IPA) software (Ingenuity Systems^®^, Redwood City, CA, USA) licensed to QIMRBerghofer.

### 2.7 siRNA transfection and cell viability

siRNA sequences as described in Table S2 were used for target validation. siRNA transfections (10 nm) were carried out using Lipofectamine^®^ RNAiMAX, and cell viability was determined using AQueous One Solution Cell Proliferation Assay kit as previously described (Al‐Ejeh *et al*., 2014; Srihari *et al*., 2016). siRNA sequences were designed as 27mers (minimizing off‐target effects compared to 21mers) and BLAST‐analyzed to check for specificity. These three sequences were pooled at a final concentration of 10–20 nm to further minimize off‐target effects (Table S2).

### 2.8 Flow cytometry

Flow cytometry analysis was performed to determine cell cycle perturbations following 6‐Gy irradiation (IR) after 6 h, fixed in 70% ethanol overnight at 4 °C, washed, and stained with propidium iodide. Cell phases were analyzed using modfit lt 4.0 software Verity (Software House, Topsham, ME, USA) (Van Schaeybroeck *et al.,* 2014).

### 2.9 Immunoblotting

Immunoblotting was performed as described previously (Srihari *et al*., 2016; Van Schaeybroeck *et al*., 2014).

### 2.10 DNA combing assay

The DNA fiber protocol was followed as described previously (Schwab and Niedzwiedz, 2011). 5 × 10^4^ cells were seeded overnight that were pulsed for 20 min with 250 μm IdU (Sigma I7125; Sigma‐Aldrich, Castle Hill, NSW, Australia), followed by pulsing with 50 μm CldU (Sigma C6891; Sigma‐Aldrich). The cells were washed thrice with PBS and trypsinized, washed once more, and resuspended in PBS. Two microlitre of cells were spotted onto glass slides (~1500 cells per slide) and lysed for 36 min with 7 μL of spreading buffer (0.5% SDS in 200 mm Tris/HCl pH 7.4 and 50 mm EDTA). Slides were tilted at a 15° angle to allow DNA spreading followed by fixation in a 3 : 1 volume of absolute methanol/glacial acetic acid for 10 min and air‐dried. Samples were denatured in 2.5 m HCl for 60 min at room temperature and rinsed before blocking in PBS/0.1% Triton X‐100/1% BSA for 1 h at room temperature. Slides were incubated for 2 h using mouse anti‐BrdU (1 : 10 dilutions; Becton, Dickinson and Company, 347580) antibody to detect IdU and anti‐BrdU (1 : 200 dilution, BU1/75; rat anti‐BrdU, Abcam, ab6326) antibody to detect CldU. Slides were then washed three times in PBS, followed by secondary antibody staining with Alexa Fluor 546‐conjugated goat anti‐mouse (1 : 300 dilutions) (Life Technologies, A‐11030) and Alexa Fluor 488‐conjugated chicken anti‐rat (1 : 300 dilutions) (Life Technologies, A21470) for 1 h at room temperature. Coverslips were mounted using Prolong Plus (Invitrogen, P36930) and imaged using a 63× objective on a Zeiss 780‐NLO confocal microscope (Zeiss, Oberkochen, Germany). Progressive replication fork speed was calculated based on the length of the CldU tracks measured using imagej software (https://imagej.nih.gov/ij/index.html). At least 300 replication tracks were analyzed for each sample in two independent experiments. The fork speed was calculated based on conversion factor 1 μm = 2.59 kb (Henry‐Mowatt *et al*., 2003).

### 2.11 Foci formation and immunofluorescence staining

Cells were seeded on the 0.1% poly‐l‐lysine‐coated coverslips or Ibidi 8‐well chamber slides. To determine RAD51/53BP1 and γH2AX foci accumulation, cells were irradiated (IR) with indicated doses of ionizing radiation using a (^137^cesium) source, pre‐extracted with cytoskeletal buffer (CSK) (10 mm Pipes, pH 6.8, 300 mm sucrose, 100 mm NaCl, 3 mm MgCl_2_, 1 mm EGTA) for 5 min, and fixed in 4% paraformaldehyde prior at the indicated time points as described previously. Foci were imaged on a Deltavision Personal deconvolution microscope as described previously (Richard *et al*., 2008) and scored using the Find Maxima function on Image J (NIH).

### 2.12 DNA repair kinetics foci formation and immunofluorescence staining

To obtain DNA repair kinetics using γ‐H2AX or 53BP1 foci, cells were irradiated, fixed at time points ranging from 0.5 to 24 h postirradiation with 1 Gy, and immunostained with indicated antibodies. The number of γ‐H2AX or 53BP1 foci was determined for each time point (average of 50 nuclei) and, after subtracting background (number of foci in nonirradiated nuclei), the percentage foci remaining was plotted against repair time to obtain DNA repair kinetics.

### 2.13 Homologous recombination repair (HRR) assay

0.5 × 10^6^ cells of the isogenic colorectal cancer lines, HCT116, HKh‐2, HKe‐3, were transiently transfected with 3 μg pDRGFP, a truncated eGFP plasmid with 1 μg pCBASceI, a vector that expresses *I‐SceI* nuclease according to a published protocol with minor modification (Pierce *et al*., 1999). Twenty‐four hour post‐transfection, cells were collected by trypsinization, resuspended in 400 μL of 1% PBS/FCS, and immediately analyzed using BD FACSCanto™ II Cell Analyzer (BD Biosciences, San Jose, CA, USA). Flow cytometry analysis was performed by excitation of eGFP with a 488‐nm laser, and emissions were collected with a 530/30 filter. For transfection efficiency and data normalization, eGFP empty vector‐transfected cells were used.

### 2.14 Statistical analysis

Student's *t*‐tests, one‐way or two‐way ANOVA with Bonferroni *post hoc* testing was performed using graphpad prism v6.0 (GraphPad Software, LaJolla, CA, USA), and the *P*‐values were calculated as indicated in figure legends. Asterisks indicate significant differences (*P < 0.05; **P < 0.01; ***P < 0.001; ****P < 0.0001), n.s. = not significant.

## 3 Results

### 3.1 Homologs of mammalian Ras in *S. cerevisiae* (Ras1 and Ras2) identify putative candidates of Ras genetic interacting genes in humans

In order to identify synthetically lethal Ras genetic interactions, we took advantage of a recently published dataset in yeast *S. cerevisiae* by Costanzo *et al*. (2010). The authors applied a stringent genetic interaction cutoff based on confidence threshold [|ε (epsilon)| < −0.08, *P *< 0.05, defined as genetic interaction score (GIS)] to determine false‐negative and false‐positive rates of genetic interactions among individual genes from 5.4 million gene–gene pairs for synthetically lethal genetic interactions. By employing this largest dataset, Deshpande *et al*. (2013) stratified 24 205 interactions among 1666 yeast *S. cerevisiae* genes with orthologs to human genes to study genetic interactions in humans. Using this filtered dataset, we were particularly interested in looking for possible genetic interactions of yeast Ras1 and Ras2 which are orthologs to human *RRAS2* and *RRAS*, respectively. The lack of colorectal cancer‐associated *RRAS* or *RRAS2* mutations led us to search for additional human orthologues of yeast *Ras1* and *Ras2*. The N‐terminal regions of human Ras proteins undergo frequent colorectal cancer‐associated hotspot mutations at codons 12, 13, and 61, and this region does share homology with yeast *Ras1* and *Ras2* (Fig. S1A) and is closely related to *KRAS* (Fig. S1B). *Ras1* and *Ras2* genes are essential for vegetative growth in yeast and share many functional similarities between the yeast and mammalian Ras genes (DeFeo‐Jones *et al*., 1985), so we reasoned that the genetic interactions with *Ras1* and *Ras2* in yeast may correspond to a genetic interaction with mammalian *KRAS*.

To test this, we applied a similar filter as Deshpande *et al*. (2013), but restricted our analysis to (a) intermediate interactions as our analysis is based on homologs and not orthologs (ε < −0.08) for both genetic interactions of gene A–gene B and *vice versa* (AB‐BA), and (b) only 1‐1 orthologs are considered. We recovered 39 putative *Ras1* and *Ras2* interacting genes in yeast (Table 1). Of these 39 putative genetic interacting genes, six orthologs to human genes (*EMG1*,* MRE11A*,* ORC2L*,* ACOT8*,* LSM6*, and *DDX31*) particularly showed strong GIS with either *Ras1* or *Ras2*, ε < −0.2, *P* < 0.05 (Table 1). Eleven orthologs genes showed intermediate GIS with ε < −0.1, while the rest were between ε < −0.1 and −0.08 GIS. Collectively, we identified a list of putative yeast Ras genetic interacting genes that could be tested for synthetic lethal interactions with Ras oncoproteins in the mammalian system.

**Table 1:** Synthetic lethal partner with RAS1/2 in yeast identifies putative synthetic lethal partners of RAS in human genome

Yeast RAS	GI	Human ortholog	Gene description	Epsilon (GIS)
RAS1	RFC3	RFC5	Replication factor C (activator 1) 5, 36.5 kDa	−0.081
RAS2	UBX5	UBXN7	BX domain protein 7	−0.081
RAS2	RAD18	RAD18	RAD18 E3 ubiquitin protein ligase	−0.082
RAS2	RPS16A	RPS16	Ribosomal protein S16	−0.083
RAS2	PDB1	PDHB	Pyruvate dehydrogenase (lipoamide) beta	−0.085
RAS2	TAN1	THUMPD1	THUMP domain containing 1	−0.085
RAS2	RPL37A	RPL37	Ribosomal protein L37	−0.094
RAS2	ELP2	ELP2	Elongator acetyltransferase complex subunit 2	−0.095
RAS2	NUP57	NUP54	Nucleoporin 54 kDa	−0.096
RAS2	PEX10	PEX10	Peroxisomal biogenesis factor 10	−0.096
RAS2	PEX14	PEX14	Peroxisomal biogenesis factor 14	−0.096
RAS1	ALG1	ALG1	ALG1, chitobiosyldiphosphodolichol beta‐mannosyltransferase	−0.102
RAS2	PAT1	PATL1	Protein associated with topoisomerase II homolog 1 (yeast)	−0.103
RAS2	VPS41	VPS41	Vacuolar protein sorting 41 homolog (*Saccharomyces cerevisiae*)	−0.104
RAS1	MMF1	HRSP12	Heat‐responsive protein 12	−0.109
RAS2	UBI4	UBC	Ubiquitin C	−0.111
RAS2	RPS19B	RPS19	Ribosomal protein S19	−0.118
RAS2	KTI12	KTI12	KTI12 chromatin‐associated homolog	−0.127
RAS2	PCP1	PARL	Presenilin‐associated, rhomboid‐like	−0.129
RAS2	SLM3	TRMU	tRNA 5‐methylaminomethyl‐2‐thiouridylate methyltransferase	−0.13
RAS2	MPP10	MPHOSPH10	M‐phase phosphoprotein 10 (U3 small nucleolar ribonucleoprotein)	−0.13
RAS2	RAD51	RAD51	RAD51 recombinase	−0.135
RAS2	RPS16B	RPS16	Ribosomal protein S16	−0.136
RAS2	PHB2	PHB2	Prohibitin 2	−0.136
RAS1	DYS1	DHPS	Deoxyhypusine synthase	−0.139
RAS2	RAD27	FEN1	Flap structure‐specific endonuclease 1	−0.139
RAS2	VPS28	VPS28	Vacuolar protein sorting 28 homolog (*S. cerevisiae*)	−0.139
RAS2	ERS1	CTNS	Cystinosin, lysosomal cystine transporter	−0.14
RAS2	TMA22	DENR	Density‐regulated protein	−0.152
RAS2	PAA1	AANAT	Aralkylamine *N*‐acetyltransferase	−0.159
RAS2	OST2	DAD1	Defender against cell death 1	−0.162
RAS2	YLR050C	TMEM97	Transmembrane protein 97	−0.166
RAS2	RIA1	EFTUD1	Elongation factor Tu GTP‐binding domain containing 1	−0.192
RAS2	EMG1	EMG1	EMG1 N1‐specific pseudouridine methyltransferase	−0.205
RAS2	MRE11	MRE11A	MRE11 homolog A, double‐strand break repair nuclease	−0.206
RAS2	ORC2	ORC2L	Origin recognition complex, subunit 2	−0.222
RAS2	TES1	ACOT8	Acyl‐CoA thioesterase 8	−0.243
RAS2	LSM6	LSM6	LSM6 homolog, U6 small nuclear RNA and mRNA degradation associated	−0.254
RAS2	DBP7	DDX31	DEAD (Asp‐Glu‐Ala‐Asp) box polypeptide 31	−0.283
aRAS1	RAS2	RRAS2 RRAS	Related RAS viral (r‐ras) oncogene homolog	−0.822
aRAS2	RAS1	RRAS2 RRAS	Related RAS viral (r‐ras) oncogene homolog 2	−0.822

GIS is genetic interaction score in yeast with *P *< 0.05.

a Known genetic interaction in yeast as positive control.

### 3.2 Ras1 and Ras2 genetic interactions in yeast are enriched in DNA damage signaling

**Figure 1:**
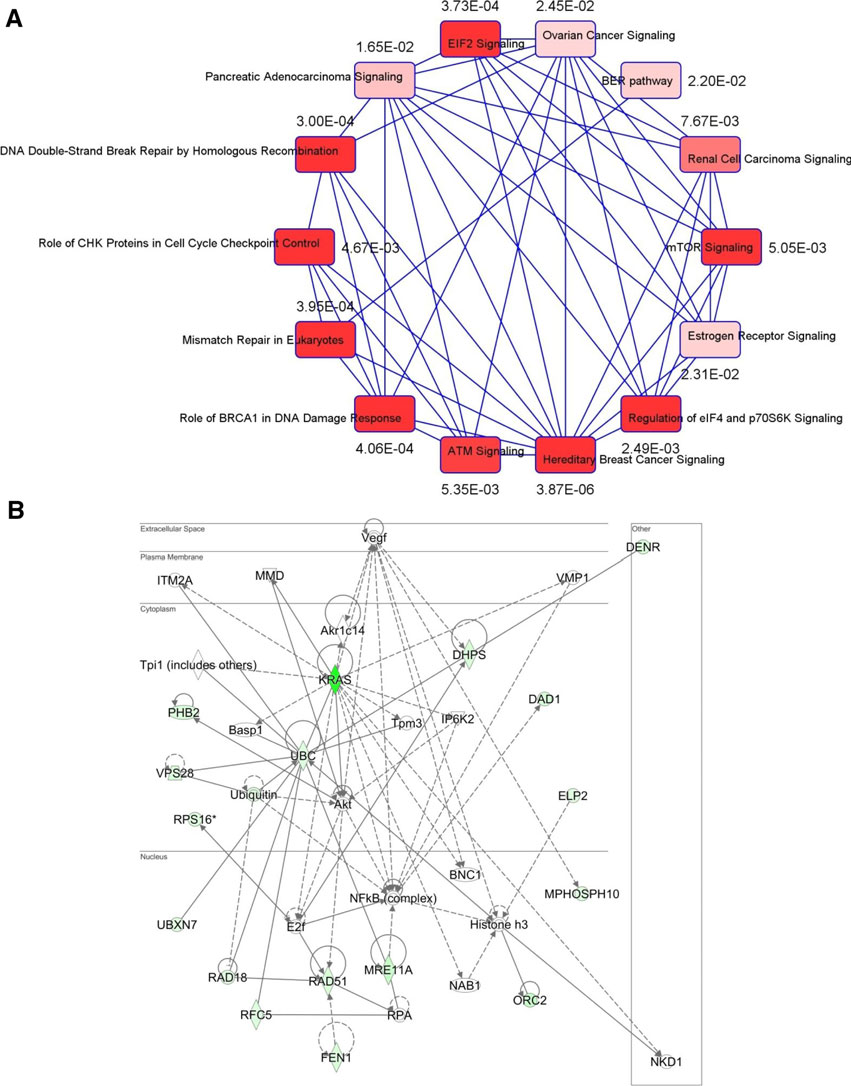
*KRAS*‐dependent genetic network analysis. (A) Correlation‐based network with *P‐*value connecting possible Ras‐interacting genes with similar genetic interaction clusters showing pathway enrichment in DNA damage response signaling. (B) Using *KRAS* as a ‘query gene’, the 39 genes were included to the ingenuity pathway analysis (IPA) to determine possible *KRAS*‐mediated network. Seventeen of 39 genes were clustered by *KRAS* gene in the network. The pathway analysis revealed central nodes of AKT or NFκB in regulating HRR genes.

Because clusters of genes from the same pathway or similar biological processes tend to share similar genetic interaction profiles (Tong *et al*., 2004), we analyzed the 39 identified genes from the Ras1 and *Ras2* interactome to determine core biological processes that are important in Ras‐mediated oncogenesis in humans. Ingenuity pathway analysis showed that multiple cancer‐mediated signaling networks clustered (Fig. 1A). Importantly, we found that this large network is substantially linked to DSB repair by HRR (*P* = 3.0 × 10^−4^), the *BRCA1*‐mediated DDR (*P* = 4.06 × 10^−4^), mismatch repair in eukaryotes (*P* = 3.95 × 10^‐4^) as well as E1F2 signaling (*P* = 3.73 × 10^−4^) (Fig. 1A), consistent with a recently published report on wild‐type (WT) *HRAS* and *NRAS* promoting mutant *KRAS*‐driven tumorigenesis by modulating the DDR in colorectal cancer cells (Grabocka *et al*., 2014). Notably, deregulated Ras signaling has been shown to compromise DNA damage checkpoint recovery in *S. cerevisiae* (Wood and Sanchez, 2010). It is also well documented that Ras signaling is hyperactivated in a majority of breast tumors (Schubbert *et al*., 2007), although mutations in Ras members are very rare in breast cancers. Consistent with this, we also noticed that the hereditary breast cancer signaling network (also enriched with DDR) (*P* = 3.87 × 10^−6^) was significantly associated with Ras signaling (Fig. 1A). Moreover, pathway enrichment analysis with *KRAS* as a query gene identified a core biological network linking 17 of 39 genes from this yeast genetic interactome to Ras‐dependent signaling either through AKT or through NFκB complexes (Fig. 1B). Both pathways have been well studied in the context of Ras signaling in cancers (McCubrey *et al*., 2012; Samatar and Poulikakos, 2014).

### 3.3 RAD51 and PARL are putative essential genes for mutant KRAS‐dependent colorectal cancer cell survival

**Figure 2:**
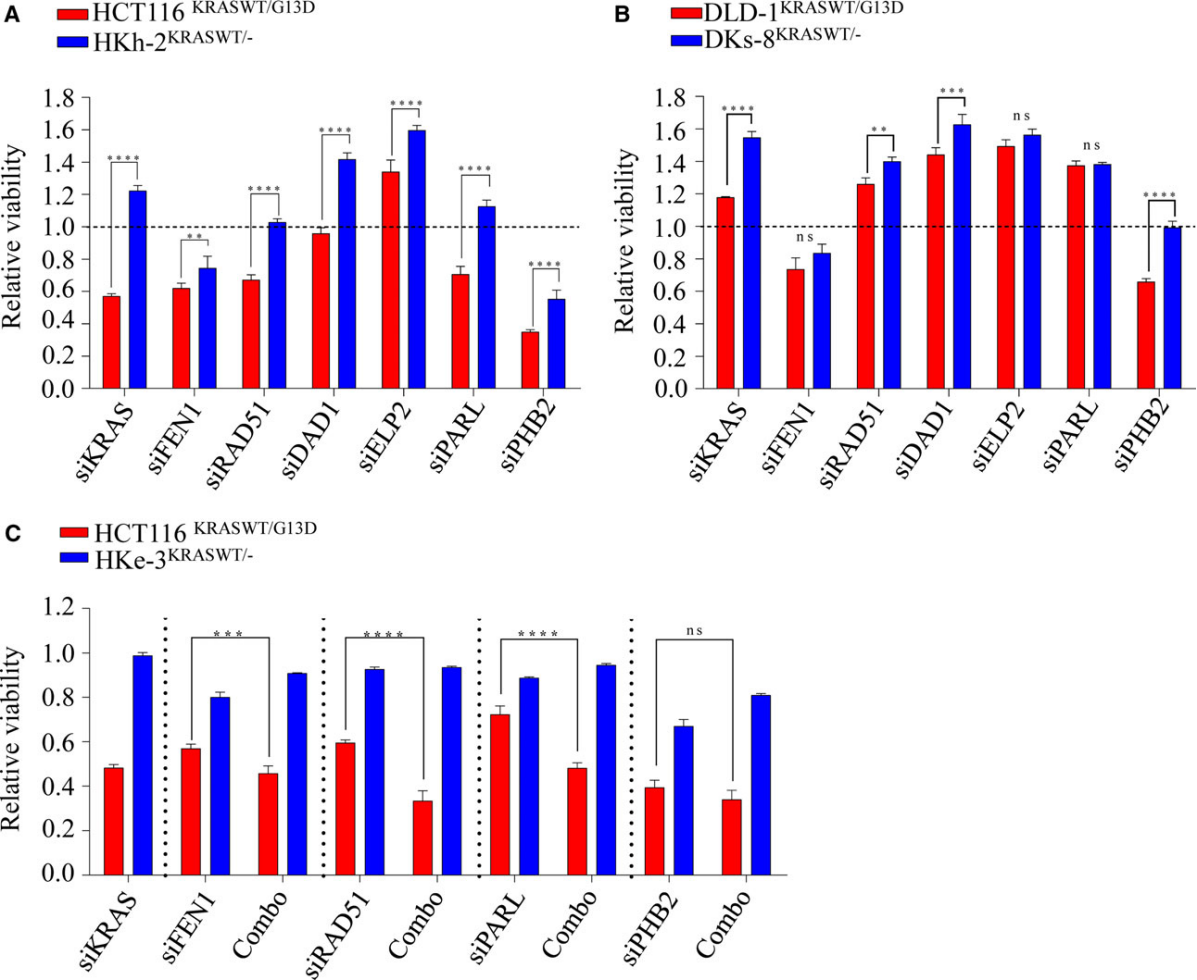
Validation of synthetic lethal interactions with *KRAS* in isogenic colorectal cancer lines. (A, B) Isogenic lines were reverse‐transfected with 10 nm of indicated siRNAs, and cell viability was determined after 96 h. (C) HCT116 and HKe‐3 lines were transfected with 10 nm of siRNA individually or in combination with siKRAS and cell viability was determined after 96 h. Scr: Scrambled siRNA was used as control and relative cell viability was determined to the scr control‐transfected cells. ****P < 0.0001; ***P < 0.001; **P < 0.01; ns: not significant. Error bars represent the standard error of the mean (SEM) from three independent experiments.

We next asked whether human Ras genes could have analogous synthetically lethal genetic pairs as yeast Ras. If this were the case, we would expect inhibition of these interactions to selectively kill mutant, constitutively activated *KRAS*‐dependent cells compared to WT counterparts. To investigate this, we utilized an isogenic set of *KRAS*‐mutant (mt) HCT116 (constitutively active KRAS) and genetically corrected WT HKh‐2 and HKe‐3 colorectal cancer lines (Shirasawa *et al*., 1993; Van Schaeybroeck *et al*., 2014), which exhibited similar growth rates (Fig. S2A). Because our network analysis was enriched with DNA damage and repair signaling, we selected *RAD51* and *FEN1* as candidate genes (both interconnected by AKT signaling; moreover, RAD51 is an essential component for HRR) (Baumann and West, 1998; Kikuchi *et al*., 2005) along with Prohibition 2 (*PHB2*)/RAF which has been shown to interact with C‐RAF for epithelial migration (Chowdhury *et al*., 2014; Mishra *et al*., 2005; Rajalingam and Rudel, 2005; Rajalingam *et al*., 2005). We also selected *DAD1*, identified as protector of apoptosis (Hong *et al*., 2000); *ELP2*, a necessary component for functional elongator (Dong *et al*., 2015), and *PARL*, an inner mitochondrial membrane rhomboid (Cipolat *et al*., 2006) as putative Ras synthetic lethal genes. RNAi‐mediated depletion of each gene in the isogenic lines showed that depletion of *RAD51* and *PARL* selectively killed *KRAS*‐dependent mutant HCT116 cells compared to WT HKh‐2 cells (Fig. 2A). This cell line is dependent on mutant KRAS for survival as *KRAS* depletion alone promotes cell death as previously reported by us and others (Steckel *et al*., 2012; Van Schaeybroeck *et al*., 2014). Unexpectedly, depletion of *DAD1* and *ELP2* significantly enhanced cell viability in *KRAS* WT cells, suggesting that mutant *KRAS* is unlikely to regulate either of these genes. Moreover, we found that depletion of *PHB2* had a more profound effect on lethality in *KRAS*‐mutant cells than their WT counterpart; however, *PHB2* depletion alone slightly reduced cell viability in WT cells (Fig. 2A). In addition, a significant differential effect was also observed following *FEN1* depletion. To confirm this selectivity, we used *KRAS*‐independent isogenic DLD‐1/DKs‐8 lines (Singh *et al*., 2012) and found that only *PHB2* depletion showed selectivity in killing mutant cells, while *FEN1* showed a similar trend of inhibition as seen in isogenic HCT116 cells (Fig. 2B). To further validate gene‐to‐gene interactions, we codepleted *KRAS* with *FEN1*,* RAD51*,* PARL*, and *PHB2* in the HCT116 and HKe‐3 pair (Figs 2C and S2B,C). We found that when *KRAS* was codepleted with *RAD51*,* FEN1*, and *PARL* (but not *PHB2*), cell viability was significantly reduced in *KRAS*‐mutant cells compared to WT cells. Collectively, our cross‐species validation of synthetic lethal interactions suggested that *RAD51*,* FEN1*, and *PARL* are likely essential in the presence of mutant KRAS‐driven signaling in colorectal cancer cells and, of these three, RAD51 is dysregulated in many cancers and has been identified as a potential target for drug discovery (Ward *et al*., 2015).

### 3.4 KRAS mutation induces replication stress and DNA damage

**Figure 3:**
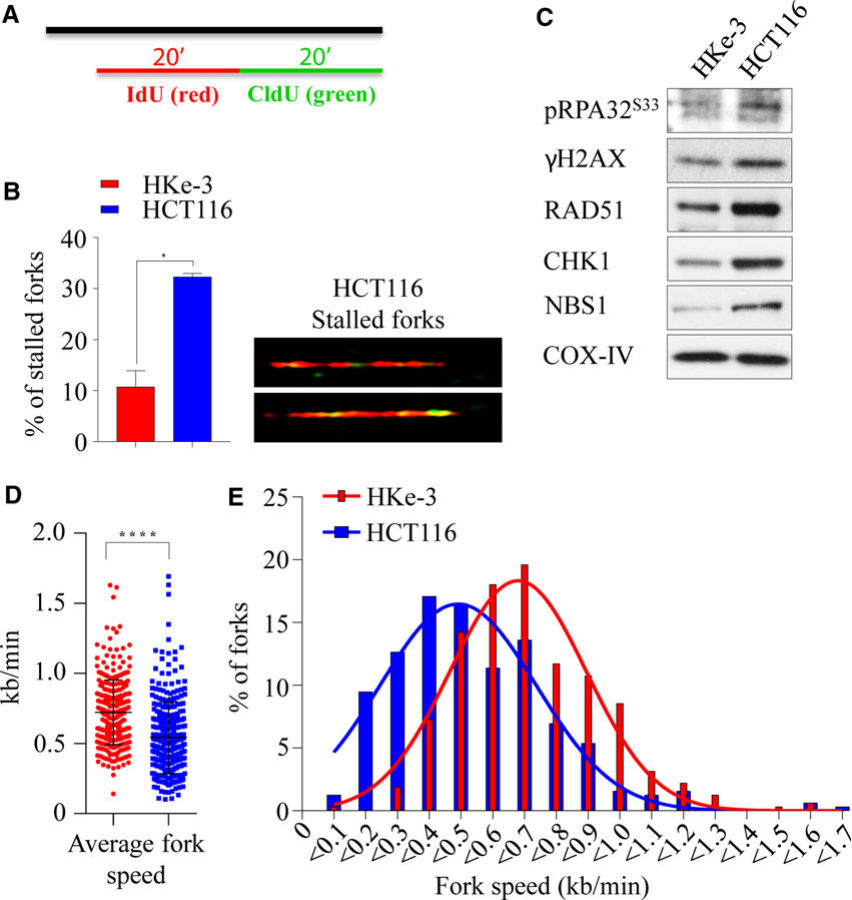
Mutant KRAS‐induced replication stress and DNA damage. (A) Labeling of BrdU analogs for DNA fiber analysis during replication. Both isogenic KRAS‐mutant (HCT116) and wild‐type (HKe‐3) cells pulse‐labeled with IdU (red) and CldU (green) for 20 min and the fibers were imaged and quantified. (B) Percentage of stalled replication forks (left) and representative images of stalled replication fork DNA fibers in HCT116 (right). (C) Immunoblot analysis of phospho‐S33 RPA32, γH2AX, RAD51, CHK1, and NBS1 in isogenic lines. COX‐IV expression was used as a loading control. Velocity of progressing forks (D) and distributions of replication fork speeds (E) were determined in both isogenic lines. At least 300 fibers from each cell line were analyzed from two independent experiment, and error bars represent the standard error of the mean (SEM). Unpaired *t*‐test with and without Welch's correction between two groups was used to determine the statistical significance, *P < 0.05; ****P < 0.0001.

Depletion of *RAD51* in isogenic *KRAS*‐mutant and WT lines as measured by both cell viability and death implicated its critical role in survival in the *KRAS*‐mutant context (Fig. S2D). The RAD51 protein is a key component of HRR‐mediated DSB repair, which occurs predominantly during the S and G2 phases of the cell cycle. As our results show that cells expressing mutant KRAS are likely dependent on RAD51 for survival, we hypothesized that these cells are reliant on augmented HRR, perhaps in order to survive a higher burden of oncogenic‐mediated replication stress and associated DNA breaks. Consistent with this notion, we found the *KRAS*‐mutant cells have a higher burden of replication stress, which is evident by a significant increase in stalled replication forks (Fig. 3A,B), which corresponded with increased γH2AX and pRPA32 (S33) signaling. Moreover, we found an increase in basal protein levels of HRR proteins including CHK1, NBS1, and RAD51 in the HCT116‐mutant line compared to the HKe‐3 WT line (Fig. 3C), which is likely to be transcriptional as per a recent report (Kotsantis *et al*., 2016). In this study, when HRAS^V12^ was ectopically overexpressed in immortalized human fibroblasts, RNA synthesis was found to be increased together with R‐loop accumulation, which resulted in replication fork slowing and DNA damage in a mutant RAS‐dependent manner. In line with this, we found that the mutant HCT116 line has a slower rate of replication fork progression compared to the WT line (Figs 3D,E and S2E). Collectively, these results suggest that mutant KRAS increases replication stress and DNA damage resulting in a greater dependency on HRR for survival.

### 3.5 RAD51 as a potential therapeutic target in KRAS‐mutant colorectal cancer

**Figure 4:**
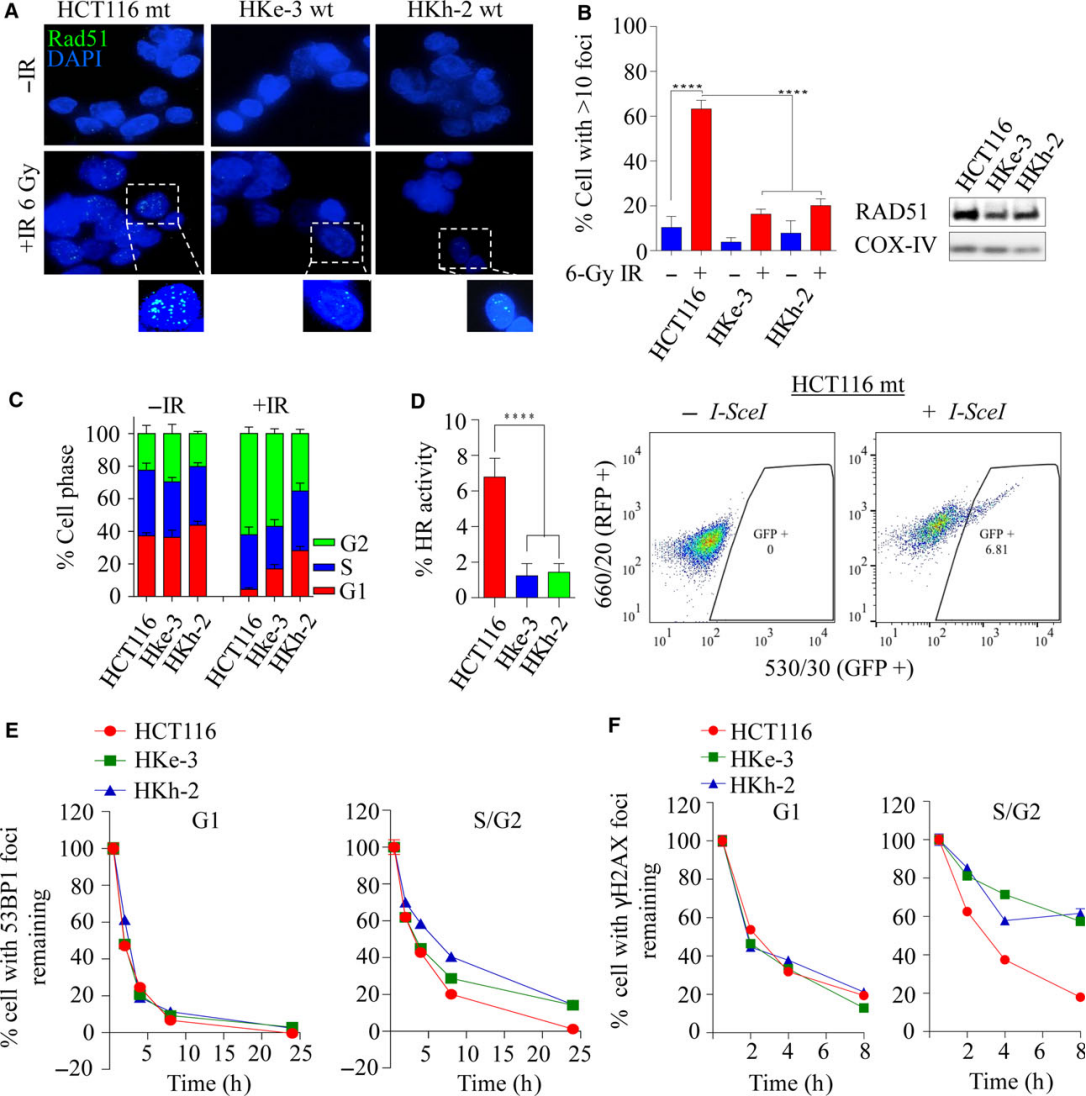
Mutant *KRAS*‐mediated RAD51‐dependent homologous recombination repair is elevated in *KRAS*‐mutant colorectal cancer. (A) Representative images of the isogenic colorectal cancer lines immunostained with anti‐RAD51 (green) and anti‐DAPI (blue) following 6‐Gy ionizing radiation processed after 6 h. (B) Left: quantification of RAD51 foci‐positive cells from experiment in A. The percentage of cells with > 10 RAD51 foci was calculated. Error bars denote SEM (*n* = 3 with more than 50 cells scored for each independent experiment). Right: immunoblot analysis of RAD51 expression in the isogenic lines. COX‐IV was used as a loading control. (C) Propidium iodide (PI) cell cycle profiles of isogenic cell lines before and at 6 h following 6 Gy of ionizing radiation, *n* = 3 ± SEM. (D) Left: HRR efficiency in isogenic lines was determined using an HRR reporter assay based on pDR‐GFP and *I‐SceI* (pCBSCE) plasmids. Twenty‐four hours after cotransfection, FACS analysis was carried out to detect GFP‐positive cells, *n* = 2 ± SEM. Right: representative images of GFP‐positive cells that had successfully undergone HRR in HCT116 cells. Quantification of (E) 53BP1 and (F) γH2AX foci clearance following 1 Gy of ionizing radiation in a time‐dependent manner in *KRAS*‐mutant and wild‐type isogenic colorectal cancer lines. *Y*‐axis represents percentage of foci remaining at the indicated time points (with the average number of foci at 0.5 h being 100%). Cells were costained for cyclin A to demarcate G1 (cyclin A −ve) versus S/G2 (cyclin A +ve) populations. The percentage of cells with foci remaining was calculated and plotted against the indicated times post‐IR. Error bars denote SEM (50 nuclei were scored for each time point).

As KRAS‐mutant cells are dependent on HRR signaling, we first sought to determine the integrity of HRR in the isogenic lines. We treated HCT116, HKh‐2, and HKe‐3 lines with 6‐Gy of IR, and assessed RAD51 foci formation after 6 h, as a surrogate for HRR efficiency. Interestingly, we observed significantly higher numbers of RAD51 foci‐positive cells in the HCT116‐mutant line compared to the HKh‐2 and HKe‐3 WT counterparts (63% *vs*. 16–20%, *P* < 0.0001), suggesting possible increased levels of HRR dependency in *KRAS*‐mutant line (Fig. 4A,B left). The increase in RAD51 foci formation can be attributed in part to increased expression of RAD51 in KRAS‐mutant cells compared to WT counterparts (Figs 3C and 4B right). To determine whether the increased RAD51 foci formation was due to an increased proportion of cells in the S and G2 phases of the cell cycle, we performed propidium iodide (PI) cell cycle analysis on these cells. Treatment with IR did not result in apoptosis, and all three cell lines exhibited similar increases in the S/G2 population [HCT116 (96%); HKe‐3 (82%); HKh‐2 (72%)], suggesting the increase in RAD51 foci does not result from cell cycle differences (Figs 4C and S3A). Thus, these results suggested that HRR‐related events are greatly stimulated in cells expressing mutant *KRAS*. To address this possibility, we used an *in vivo* plasmid recombination GFP‐based assay, which measures reconstitution of GFP signal after induction of DSBs by the *I‐SceI* endonuclease (Pierce *et al*., 1999). As shown in Fig. 4D, *KRAS*‐mutant cells exhibited a significantly higher rate of HRR (fivefold increase) compared to isogenic WT cells. Consistent with this, we found that *KRAS*‐mutant cells were more proficient than WT cells in repairing IR‐induced DSBs, quantified by 53BP1 or γH2AX foci clearance (Figs 4E,F and S3B,C). Importantly, this increase in repair proficiency was seen only in cells in the S/G2 phases of the cells cycle where HRR is used for repair, but not in the G1 phase where nonhomologous end joining (NHEJ) is the repair pathway of choice. This suggests that *KRAS* mutation may play a significant role in DNA damage clearance during the S and G2 phases of the cell cycles and that this predominantly occurs by employing HRR.

### 3.6 RAD51 loss impacts survival advantage in KRAS‐mutant colorectal cancer

**Figure 5:**
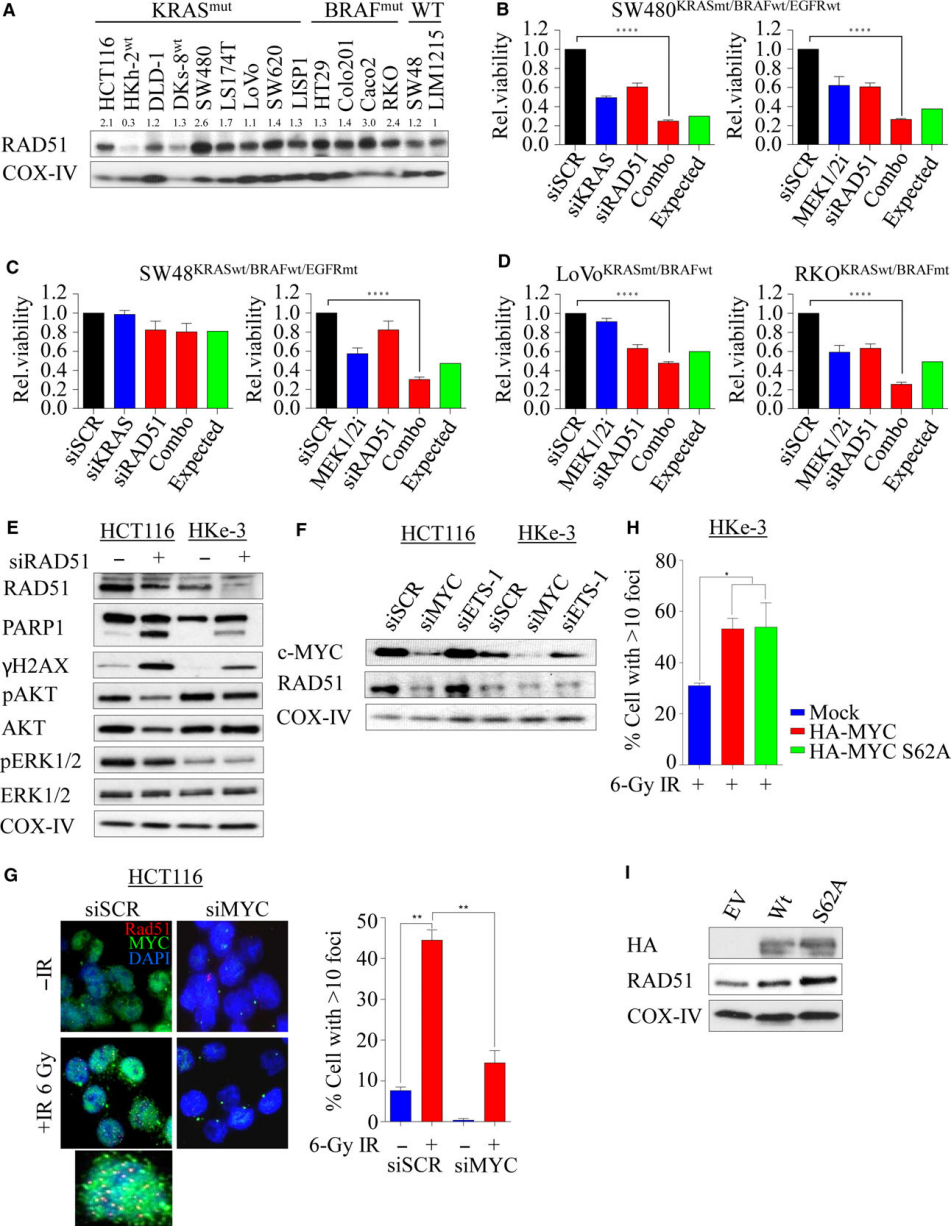
RAD51 loss impacts survival advantage in KRAS‐mutant colorectal cancer. (A) Immunoblot analysis of RAD51 expression in a panel of human colorectal cancer lines including the isogenic lines. COX‐IV expression was used as a loading control. Protein band intensities were measured using imagej software. RAD51 protein expression across the cell lines was calculated relative to KRAS wild‐type LIM1215 cell line. (B–D) A panel of colorectal cancer lines was either reverse‐transfected with 10 nm of siRNA (left) or treated with 0.5 μm AZD6244, a MEK1/2 inhibitor, and cell viability was determined after 96 h. Relative cell viability was determined by comparing with scrambled control‐transfected/DMSO‐treated cells. (E) Isogenic paired lines were reverse‐transfected with 10 nm of pooled siRNA against *RAD51*, and after 48 h, immunoblot analysis was performed to determine expression of RAD51, cleaved PARP, total and phosphorylated H2AX, AKT, and ERK1/2. COX‐IV expression was used as a loading control. (F) Immunoblot analysis of RAD51 expression following c‐MYC and ETS‐1 silencing in the isogenic lines. COX‐IV expression was used as a loading control. (G) Representative images of RAD51 foci with and without c‐MYC silencing in irradiated (6‐Gy) HCT116 cells, immunostained 6 h later with anti‐RAD51 (red), anti‐c‐MYC (green), and anti‐DAPI (blue). Right: quantification of RAD51 foci cells from experiment in F. The percentage of cells with > 10 RAD51 foci counted. Error bars denote SEM (more than 200 nuclei were scored). (H) Quantification of IR (6‐Gy)‐induced RAD51 foci‐positive cells analyzed 6 h after treatment in HKe‐3 cells transfected with either wild‐type HA‐tagged MYC or phospho‐mutant S62A. Mock‐transfected parental cells were used as a control. The percentage of cells with > 10 RAD51 foci were counted. (I) Immunoblot analysis of RAD51 expression following c‐MYC overexpression in HKe‐3 cells. COX‐IV expression was used as a loading control. Error bars denote SEM (more than 200 nuclei were scored) ****P < 0.0001; ***P < 0.001; **P < 0.01.

To determine whether increased RAD51 expression is a common phenomenon in mutant *KRAS* cell lines, we next assessed a panel of colorectal cell lines for RAD51 protein levels by immunoblotting (Fig. 5A). We noted a trend toward increased RAD51 levels in *KRAS‐* and *BRAF‐*mutant lines compared to WT lines, suggesting a possible reliance on RAD51 expression and HRR dependence. Given the apparent increase in RAD51 levels in KRAS‐ and BRAF‐mutant cell lines, we next wanted to test for their dependency between *KRAS* and *RAD51*. To investigate this, we depleted *RAD51* alone or in combination with *KRAS* by siRNA in SW480 (*KRAS*mt) and SW48 (*KRAS*wt) cell lines and found that combined depletion decreased cell viability in mutant, but not WT cells (Fig. 5B,C left). To further confirm this observation, we used 0.5 μm AZD6244 (Davies *et al*., 2007), a MEK1/2 inhibitor to inhibit ERK1/2‐dependent signaling (a major downstream effector of mutant *KRAS*) in these cells. MEK1/2 inhibition alone significantly affected cell viability in both cell lines irrespective of *KRAS* mutational status, and this was enhanced following RAD51 depletion (Fig. 5B,C right). One possible explanation for this synergistic effect observed in SW48wt could be due to an *EGFR* mutation (c.2155G>A) which constitutively activates MAPK signaling in this line. Consistent with this, nuclear EGFR has been shown to play roles in regulating DSB repair by interacting with DNA‐PK, ATM, RAD51, and BRCA1 either through PI3K‐AKT or through Ras‐Raf‐MAPK pathway (Chen and Nirodi, 2007; Mukherjee *et al*., 2009, 2010). Moreover, Yeh *et al*. (2009) suggested that KRAS/BRAF‐independent ERK1/2 activation in WT cells may account for MEK1/2i sensitivity (Hao *et al*., 2007). Despite this, we found that the WT DKs‐8 cells did not respond to combined inhibition (Fig. S4A). In addition, *RAD51* depletion alone decreased cell viability in both LoVo (*KRAS* mt) and RKO (*BRAF* mt) lines, and this was significantly enhanced with MEK1/2 inhibition at a proportion greater than the expected lethality (Fig. 5D). In line with this, we also depleted *RAD51* in the isogenic HCT116 (mt) and HKe‐3 (WT) lines for 48 h to see whether the specific cell inhibition in mutant cells was accompanied by apoptosis. We found that mutant HCT116 cells were more susceptible to RAD51 loss‐dependent apoptosis compared to WT cells. This was associated with an increase in DSBs following RAD51 depletion marked by γ‐H2AX protein accumulation (Fig. 5E). Moreover, we found that following RAD51 depletion, survival signaling such as ERK1/2 and AKT was impacted in mutant HCT116 cells, but not in WT HKe‐3 cells, suggesting that RAD51 plays a significant role in regulating cell survival pathways through MAPK‐AKT signaling (Fig. 5E). Collectively, our data suggest that *KRAS‐* or *BRAF*‐mutant colorectal cancer lines are likely selectively dependent on Ras‐mediated *RAD51‐*dependent HRR signaling for survival.

### 3.7 c‐MYC‐mediated RAD51‐dependent HRR is essential in KRAS‐mutant colorectal cancer cells

Upon growth stimulation, Ras triggers a complex array of signal transduction that activates a number of cellular pathways involved in cell proliferation and survival regulated by MAPK and PI3K/AKT networks. Hyperactivation of these pathways through KRAS‐dependent ERK1/2 and/or AKT/mTOR leads to aberrant c‐MYC transcriptional activity during tumorigenesis (Sears *et al*., 2000). It has been shown through chromatin immunoprecipitation studies that MYC can bind to promoters of a number of DDR genes including RAD51 (Luoto *et al*., 2010; Mao *et al*., 2003). c‐MYC is an ERK substrate (Hayes *et al*., 2016; Wu *et al*., 2007), and in line with this, RAS mutation in HCT116 cells significantly enhanced basal c‐MYC protein levels (Fig. 5F). In order to mechanistically show the role of RAS signaling in promoting HRR through upregulation of RAD51, we explored the effect of *MYC* depletion on RAD51 protein expression. Depletion of *MYC* in both HCT116 and HKe‐3 cells caused a significant reduction in RAD51 protein levels (Fig. 5F). As a comparison, we also depleted *ETS‐1*, another ETS transcription factor downstream of ERK signaling (Foulds *et al*., 2004; Ohtani *et al*., 2001), and found no changes in RAD51 protein levels. When we challenged both control and *MYC*‐depleted *KRAS*‐mutant cells with IR, we found significantly fewer RAD51 foci in MYC‐depleted cells compared to sicontrol cells (*P* < 0.01; Fig. 5G), while c‐MYC depletion had no effect in *KRAS* WT cells (Fig. S4B). Phosphoserine 62 (pS62) and threonine 58 (pT58) are two essential phospho‐sites that control c‐MYC stability and degradation, respectively (Sears, 2004). The former is phosphorylated by ERK and CDKs, while the latter is phosphorylated by GSK3β which triggers SCF‐Fbw7‐dependent ubiquitination and degradation. To explore the role of c‐MYC activation in promoting RAD51‐dependent DNA repair, we ectopically overexpressed HA‐tagged WT c‐MYC and phospho‐mutant S62A in the KRAS WT cell line HKe‐3. Constitutive induction of WT and stable S62A c‐MYC significantly increased IR‐induced RAD51 foci (Fig. 5H) with a corresponding increase in RAD51 protein levels (Fig. 5I). Collectively, our data show a direct link between constitutive KRAS signaling in regulating HRR through c‐MYC during the DDR.

### 3.8 Combined CHK1‐PARP1 inhibition synergistically induces cell death in KRAS‐mutant colorectal cancer cells

**Figure 6:**
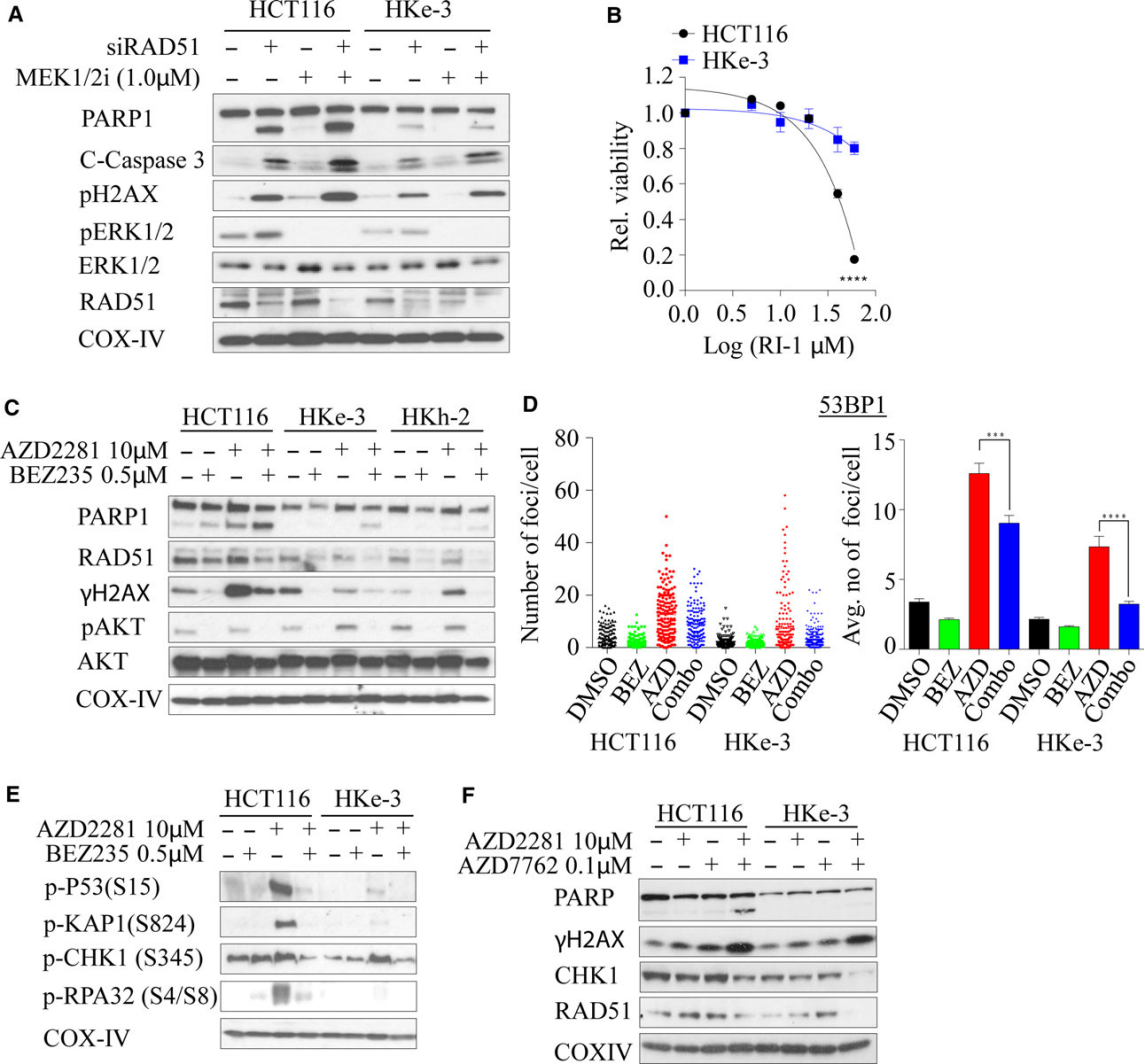
Dual inhibition of RAD51‐MEK1/2, PI3K/Akt/mTOR‐PARP1, or CHK1‐PARP synergistically induces lethality in *KRAS‐*mutant colorectal cancer cells. (A) Isogenic *KRAS‐*mutant colorectal cancer lines were reverse‐transfected with 10 nm of si*RAD51* for 24 h, followed by 1.0 μm of AZD6244, and immunoblot analysis was performed for an additional 24 h to determine the expression of RAD51, cleaved PARP, γH2AX, total and phosphorylated ERK1/2. (B) Isogenic colorectal cancer cell lines were exposed with different concentrations of RI‐1, a RAD51 inhibitor, and cell viability was determined after 96 h. The dose–response curve was generated by calculating relative cell viability plotted against drug concentration. (C) Isogenic *KRAS‐*mutant colorectal cancer lines were treated with 10 μm of AZD2281, a PARP inhibitor alone, or in combination with 0.5 μm BEZ235, a PI3K/Akt/mTOR inhibitor, and immunoblot analysis was performed for 48 h to determine the expression of RAD51, cleaved PARP, γH2AX, and phosphorylated AKT. (D) *KRAS‐*mutant isogenic colorectal cancer lines were treated with BEZ235, AZD2281, or a combination of both inhibitors for 24 h and immunostained for 53BP1 foci. Individual foci number counts (left panels) and average number of foci per cell (right panel) are shown. Error bars denote SEM (*n* = 2 where more than 50 cells were scored for each experiment). (E) Isogenic *KRAS‐*mutant colorectal cancer lines were treated with 10 μm of AZD2281 alone or in combination with 0.5 μm BEZ235, and immunoblot analysis was performed for 48 h to determine phosphorylation of p53 (S15), KAP1 (S824), CHK1 (S345), and RPA32 (S4/S8). (F) Isogenic *KRAS‐*mutant colorectal cancer lines were treated with 10 μm of PARP1 inhibitor alone or in combination with 0.1 μm AZD7762, a CHK1 inhibitor, and immunoblot analysis was performed for 48 h to determine the expression of RAD51, cleaved PARP, γH2AX, and CHK1. COX‐IV expression was used as a loading control.

Because RAD51 has a central role in HRR‐ and *KRAS*‐mutant cells that are selectively dependent on RAD51 for survival, we depleted *RAD51* for 24 h followed by MEK1/2 inhibition (to inhibit MAPK signaling) in HCT116/HKe‐3 cells. We found that coinhibition of RAD51 and MEK1/2 signaling induced DNA damage and apoptosis, evident by accumulation of γ‐H2AX, cleaved PARP‐1, and caspase 3 predominantly in *KRAS‐*mutant HCT116 cells compared with WT cells (Fig. 6A). To further confirm the RAD51 dependency in *KRAS‐*mutant cells, we treated both isogenic lines with the RAD51 inhibitor RI‐1 (Budke *et al*., 2012), and found that *KRAS‐*mutant cells are more sensitive to RAD51 inhibition with an EC50 of ~ 45 μm, while *KRAS* WT HKe‐3 cells showed only a limited response at the highest dose tested (Fig. 6B). Next, we treated both the isogenic lines with RI‐1 in combination with the MEK1/2 inhibitor AZD6244 and found that combined inhibition induced DNA damage and PARP‐1 cleavage in the *KRAS‐*mutant line with minimal effect on the WT line (Fig. S4C).

Because of the lack of clinically potent selective RAD51 inhibitors, as well as in order to further suppress the DSB repair in these cells, we first used NVP‐BEZ235, a dual PI3K/Akt/mTOR inhibitor and also a potent ATM and DNA‐PK inhibitor, which can inhibit both the HRR and NHEJ pathways and is currently in phase I/II clinical trials for advanced solid tumors (Mukherjee *et al*., 2012). Moreover, the PI3K/AKT/mTOR pathway is one of the major signaling pathways that Ras regulates besides MAPK signaling, and our network analysis interconnected RAD51 through AKT signaling (Fig. 1B) (Chang *et al*., 2014; Gil del Alcazar *et al*., 2014; Mukherjee *et al*., 2012). We expected that inhibition of HR and NHEJ through NVP‐BEZ235 would force cells to be more reliant on PARP1‐dependent alternative NHEJ (alt‐NHEJ), and thus, combined inhibition with PARP1 should synergistically kill mutant cells rather than WT cells. To demonstrate this, we cotreated HCT116 isogenic lines with BEZ235 and olaparib (AZD2281), a PARP inhibitor, and found that mutant cells displayed enhanced PARP‐1 cleavage in combination treatment compared to single drug treatments and combination treatment of either WT line (Fig. 6C). This was associated with a BEZ235‐induced reduction in RAD51 expression (Fig. 6C). Moreover, we found that γ‐H2AX was enhanced following olaparib treatment in mutant cells and this was reduced following dual inhibition, due to BEZ235‐mediated inhibition of DNA‐PK, and ATM reported by us previously (Mukherjee *et al*., 2012) (Fig. 6C). In line with this, we found that foci formation of both 53BP1 and γ‐H2AX after 24 h was significantly reduced following dual inhibition (Figs 6D and S4D). Moreover, through immunoblotting, we found robust post‐translational modifications of repair proteins such as phosphorylation of S15‐p53, S824‐KAP1, S345‐CHK1, and S4/S8‐RPA in KRAS‐mutant compared to WT cells following olaparib treatment and this was subsequently dampened following the addition of BEZ235 (Fig. 6E). This suggests that in the presence of olaparib, mutant cells rapidly accumulate DNA damage and addition of BEZ235 simply suppresses DNA damage signaling and the accumulation of DNA damage repair proteins at the break sites (although more breaks would have been generated following combined olaparib and BEZ235 treatment), and hence the dampened repair kinetics. Because our strategy targeted DDR signaling as a whole, we wondered whether inhibition of CHK1 kinase that is required for HRR (Sorensen *et al*., 2005) and replication stress response might selectively kill KRAS‐mutant lines in combination with PARP inhibition. Treatment of both isogenic lines with a CHK1 inhibitor AZD7762 and PARP inhibition resulted in enhanced PARP‐1 cleavage compared to WT treated lines, similar to our observation with the BEZ235‐PARPi combination treatment (Fig. 6F). Collectively, our data support these novel combination treatment options to exploit enhanced lethal vulnerability in cells harboring otherwise nondruggable *KRAS* mutations.

## 4 Discussion

Despite recent advances in personalized medicine and a better understanding of human cancers, targeting oncogenic drivers such as *KRAS* still remains a challenging issue in the cancer therapeutic armamentarium. Using cross‐species validation from the largest yeast genetic interactome (Costanzo *et al*., 2010), we provide evidence of the novel role of KRAS signaling biology in upregulating HRR and as a potential therapeutic strategy in killing *KRAS‐*mutant colorectal cancer cells.

Similar to mammalian Ras signaling, *S. cerevisiae* contains two *Ras* genes, *Ras1* and *Ras2*, which share homology to the N termini of human Ras proteins. Double mutants of *Ras1* ^‐^; *Ras2* ^‐^ are nonviable, thus showing essentiality in yeast vegetative growth (Kataoka *et al*., 1984). Convergent evolution between yeast and human Ras proteins facilitated our identification of human RAS synthetic lethal pairs by mining yeast data. Pathway analysis of these genes identified that DSB repair by HRR was specifically deregulated by mutant Ras signaling. This was associated with strong genetic interactions with *MRE11A* (Krejci *et al*., 2012) and intermediate interactions with *RAD51* (Krejci *et al*., 2012), *RAD18* (Huang *et al*., 2009), and *FEN1* (Kikuchi *et al*., 2005), all components of HRR‐mediated repair which occurs predominantly in the S and G2 phases of cell cycle.

We found that silencing of *FEN1* and *PHB2* is lethal in both *KRAS* WT and *KRAS‐*mutant cells, although there was a slight difference in cell viability between the two lines. This is not surprising given the role of FEN1 in cleaving 5′‐flaps of branched DNA structures (Harrington and Lieber, 1994). Previous reports have shown that the PHB2/RAF interaction is required for MAPK activation (Chowdhury *et al*., 2014); however, we found that WT *KRAS* cells do require PHB for cell survival, further suggesting that PHB2 is unlikely to be regulated by mutant KRAS. In contrast, we found that silencing of *PARL*, a presenilin‐associated rhomboid‐like gene, selectively reduced cell viability in *KRAS‐*mutant cells. PARL has been shown to function as an antiapoptotic regulator mainly through OPA1‐dependent mitochondria cristae remodeling. In line with this, recent data reinforce a strong interrelationship between mutant KRAS signaling and mitochondrial dysfunction (Hu *et al*., 2012).

The most lethal interaction of Ras was observed with *RAD51* depletion alone or in combination with *KRAS*. RAD51 is required for strand invasion during HRR‐mediated DSB repair. RAD51 overexpression has been linked to poor overall survival in colorectal cancer (Tennstedt *et al*., 2013) and identified as one of the most predictive factors in preoperative chemoradiation therapy for advanced rectal cancer (Iwata *et al*., 2012). Consistent with this, we observed that deletion of the mutant KRAS allele led to decreased RAD51 expression in isogenic lines, suggesting a specific role of *KRAS* mutations in inducing RAD51 expression. Consistent with this notion, it has been well established that oncogenes such as *KRAS* induce DNA replication stress and consequently activate the DDR early in tumorigenesis as a barrier to proliferation and survival (Hills and Diffley, 2014). In support of this, we found that *KRAS*‐mutant cells have more stalled replication forks than WT cells and the fork rate was significantly reduced in the mutant line. Oncogenes such as HRAS^V12^ have been shown to regulate global transcription factors to stimulate RNA synthesis and cause subsequent R‐loop formation. Interference between transcription and replication machinery can lead to replication fork slowing, increased DNA damage, and genomic instability (Bartkova *et al*., 2006; Kotsantis *et al*., 2016). Cells tend to overcome these barriers by augmenting components of checkpoint and repair machineries in order to survive.

Ras signaling plays major roles in modulating cell cycle progression, yet the link between mutant *KRAS* and DSB repair is not well established. However, the upstream receptor tyrosine kinase EGFR pathway has been well studied in the context of the DDR (Chen and Nirodi, 2007). Upon DSB induction, EGFR translocates to the nucleus and promotes repair predominantly via NHEJ and HRR (Chen and Nirodi, 2007; Kriegs *et al*., 2010; Mukherjee *et al*., 2010). During NHEJ repair, EGFR has been shown to interact with the catalytic subunit of DNA‐PK, an essential component of NHEJ (Golding *et al*., 2009) while for HRR, EGFR activity is required for BRCA1 localization and subsequent RAD51 recruitment to sites of DNA damage (Li *et al*., 2008). Consistent with this, we found that EGFR‐mutant SW48 cells showed enhanced lethality when treated with MEK1/2 inhibition in combination with RAD51 depletion. Approximately 5–10% of patients with colorectal cancer harbor BRAF mutations, which has been associated with poor clinical outcomes (Tran *et al*., 2011). We found that silencing of *RAD51* also inhibited survival of *BRAF*‐mutant cells in addition to the *KRAS‐*mutant cells, suggesting that HRR‐mediated repair is a major potentially druggable driver of oncogene‐addicted tumors.

In order to further illustrate the impact of RAD51‐mediated DSB repair on the survival of *KRAS‐*mutant cells, we depleted *RAD51* in these cells and demonstrated that depletion of *RAD51* significantly modulates survival signaling and apoptosis in *KRAS‐*mutant cells. Surprisingly, we also found that AKT signaling was impacted following RAD51 depletion. Recently, several studies have shown the involvement of AKT signaling in modulating DDR (Xu *et al*., 2012) (Fraser *et al*., 2011). AKT has been shown to promote NHEJ‐mediated repair by accumulating at the vicinity of IR‐induced DSBs and colocalizing with γH2AX and ATM‐pSer1981 (Fraser *et al*., 2011). On the other hand, overexpression of AKT subsequently suppresses ATR/CHK1 signaling and HRR‐mediated repair by indirectly inhibiting resection factors such as RPA, BRCA1, and RAD51 (Xu *et al*., 2012). This can further explain the selectivity of repair kinetics between the *KRAS* WT and *KRAS‐*mutant cells where the WT cells have higher activity of AKT signaling which may suppress HRR. Moreover, we found that silencing of RAD51 impacts MAPK signaling in a *KRAS‐*mutant‐dependent manner, and further support the hypothesis that RAD51 regulates *KRAS‐*mutant cells by modulating cell signaling cascades. Mechanistically, we showed that *MYC*, a transcriptional factor involved in many cancers (Dang, 2012) and a well‐known substrate of MAPK/ERK (Hayes *et al*., 2016; Wu *et al*., 2007), facilitates IR‐induced DSB repair through regulation of HRR in *KRAS*‐mutant cells. In line with our findings, Chen *et al*. (2011) reported c‐MYC‐mediated BRCA1 promoter activation, an essential component of HRR in breast cancer, which influenced *I‐SceI‐*induced HRR. Moreover, mapping of c‐MYC binding to various promoters of DDR genes including *RAD51* highlights the regulatory role of c‐MYC in regulating DDR in which the authors showed suppression of HRR following *MYC* depletion (Luoto *et al*., 2010).

While inhibition of constitutive ERK1/2 signaling has limited effect in *KRAS‐*mutant colorectal cancer patients due to compensatory pathway activation, we found that combined inhibition of RAD51 and MEK1/2 significantly increases apoptosis by inducing DSBs. Pharmacological inhibition of RAD51 selectively killed *KRAS‐*mutant cells, suggesting they are strongly dependent on HRR. Consistent with this, MEK1/2 inhibition in KRAS‐mutant small‐cell lung cancer cells has been correlated with enhanced radiation‐induced DNA damage compared to WT cells (Lin *et al*., 2014), suggesting a novel combination strategy of targeting Ras signaling with RAD51 and MEK1/2 inhibition. Alternatively, we also found that dampening of the DDR with BEZ235 (also inhibits both canonical NHEJ and HRR) (Chang *et al*., 2014; Mukherjee *et al*., 2012), in combination with PARP1 inhibition, is synergistic in inducing apoptosis in *KRAS‐*mutant cells, likely due to increased reliance of these cells on alternative repair pathways such as alt‐NHEJ in which PARP1 is one of the critical proteins. Consistent with this, a report suggested that *KRAS‐*mutant leukemic cells rely on alt‐NHEJ upon genotoxic stress and targeting this pathway significantly enhanced chemotherapy‐induced toxicity (Hahnel *et al*., 2014). Moreover, we also show that treatment of KRAS‐mutant cells with a CHK1 kinase inhibitor (which inhibits HRR) sensitizes these cells to PARP inhibition. Consistent with this, it has recently been reported that targeting the global stress kinase pathway p38/MK2 in combination with CHK1 significantly enhanced toxicity in a KRAS‐mutant‐dependent manner (Dietlein *et al*., 2015). In addition, a recent study has suggested that mutant KRAS requires WT HRAS and NRAS for activation of CHK1 (Grabocka *et al*., 2014). Thus, knockdown of either WT H/NRAS or CHK1 inhibition can sensitize KRAS‐mutant cells to DNA‐damaging agents.

One limitation of our study was that the majority of the work was conducted in a single isogenic paired cell line model, HCT116 and its derivatives, which is dependent upon KRAS mutation for survival. It would therefore be worthwhile to validate our observation that KRAS‐mutant‐dependent tumors are reliant upon DDR using large‐scale datasets such as TCGA and tissue microarray in future studies. However, determining the actual tumor dependency on mutant KRAS for survival from such genetic data is difficult and *in vivo* validation is required by taking into account for KRAS dependency. We could, in principle, generate a KRAS‐mutant‐dependent associated DDR signature that could be used to determine tumors that are vulnerable to DDR‐targeting agents. In addition, it is also important to note that intertumoral heterogeneity may exist with regard to DDR alterations both within a given tumor subtype and between tumor types. This heterogeneity should be carefully considered when designing therapies targeting a particular genetic background.

## 5 Conclusions

We report the importance of upregulated HRR in promoting the survival of *KRAS‐*mutant colorectal cancer cells. By studying synthetic lethal interactions, we are able to underpin the significance of altered oncogene‐induced DSB repair activity in *KRAS* mutants. By testing a range of potential therapeutic combinations, we demonstrate that analysis of compensatory pathway activation can facilitate design of synthetic lethal chemotherapeutic combinations to address *KRAS*‐mutant cancer.

## Author contributions

MK, SB, and KKK conceived and designed the experiments. MK, ALB, BM, PN, DMN, SKH, JLH, GNS, and DS performed the experiments. MK, ALB, PN, BM, and SB analyzed the data. SS and SS contributed reagents/materials/analysis tools. All authors wrote and revised the manuscript. All authors read and approved the final manuscript.

## Supporting information


**Fig. S1.** Homology sequence analysis between human and yeast Ras.
**Fig. S2.** (A) Growth kinetic of HCT116 isogenic pair lines determined using the IncuCyte ZOOM^®^ live cell imager (phase‐only processing module). The percentage of cell confluence was determined using an IncuCyte mask analyzer. (B) HCT116 Isogenic pair lines were reverse transfected with 10 nm of the pool siRNA as indicated and mRNA levels were determined relative to scramble control siRNA transfected cells. Error bars represent the standard deviation of the mean from triplicates. (C) Colorectal cancer lines were reverse transfected with 10 nm of the pool siRNA of RAD51 and immunoblot was performed to determine RAD51 protein levels. (D) HCT116 Isogenic pair lines were reverse transfected with 10 nm of the pool siRNA of RAD51 for 96 h and cell viability and death were determined using MultiTox Glo multiplex cytotoxicity assay. Scr: scrambled siRNA was used as control and relative cell viability was determined to the scr control transfected cells. *P < 0.05; **P < 0.01; ns: not significant. Error bars represent the standard error of the mean (SEM) from three independent experiments. (E) Representative images of replication fork DNA fibers in both wild‐type and mutant KRAS lines.
**Fig. S3.** (A) Representative cytogram images of cell cycle distribution analyzed at 6 h after exposure to IR (6 Gy) in the isogenic lines using ModFit LT 4.0 software. (B) Percentage of cell cycle distribution analyzed at 24 h following 1 Gy of IR treatment in the isogenic lines using ModFit LT 4.0 software. *n* = 2 ± SEM. (C) Representative images of the isogenic colorectal cancer lines coimmunostained with anti‐53BP1 (green), Cyclin A (red) and DAPI (blue) following 1 Gy ionizing radiation processed after 0.5 or 24 h.
**Fig. S4.** (A) DKs‐8 cells were reverse transfected with either 10 nm of siRNA (left) or treated with 0.25 μm AZD6244, a MEK1/2 inhibitor and cell viability was determined after 96 h. Relative cell viability was determined by comparing with scrambled control transfected/DMSO treated cells. (B) Quantification of IR (6 Gy)‐induced RAD51 foci‐positive cells analyzed at 6 h after treatment in c‐MYC‐depleted HKe‐3 cells. siSCR transfected parental cells were used as a control. The percentage of cells with > 10 RAD51 foci were counted. *n* = 2 ± SEM. (C) Isogenic *KRAS‐*mutant colorectal cancer lines were treated with 1.0 μm of MEK1/2i (AZD6244) alone or in combination with 25 μm RI‐1 and immunoblot analysis was performed after 48 h to determine the expression of cleaved PARP, RAD51, γH2AX and phosphorylated ERK1/2. COX‐IV expression was used as a loading control. (D) *KRAS‐*mutant isogenic colorectal cancer lines were treated with BEZ235, AZD2281 or a combination of both inhibitors for 24 h and immunostained for γH2AX foci.
**Table S1.** Primer sequences used in this study.
**Table S2.** siRNA sequences used in this study.
