# Supplementary material for: Enhanced dependency of KRAS‐mutant colorectal cancer cells on RAD51‐dependent homologous recombination repair identified from genetic interactions in Saccharomyces cerevisiae
Source: Mol Oncol. 2017 Mar 27;11(5):470–90. doi: 10.1002/1878-0261.12040 (PMC5527460; doi:10.1002/1878-0261.12040)
Supplement: Supplementary file 1 — Fig. S1. Homology sequence analysis between human and yeast Ras. Fig. S2. (A) Growth kinetic of HCT116 isogenic pair lines determined using the IncuCyte ZOOM® live cell imager (phase‐only processing module). The percentage of cell confluence was determined using an IncuCyte mask analyzer. (B) HCT116 Isogenic pair lines were reverse transfected with 10 nm of the pool siRNA as indicated and mRNA levels were determined relative to scramble control siRNA transfected cells. Error bars represent the standard deviation of the mean from triplicates. (C) Colorectal cancer lines were reverse transfected with 10 nm of the pool siRNA of RAD51 and immunoblot was performed to determine RAD51 protein levels. (D) HCT116 Isogenic pair lines were reverse transfected with 10 nm of the pool siRNA of RAD51 for 96 h and cell viability and death were determined using MultiTox Glo multiplex cytotoxicity assay. Scr: scrambled siRNA was used as control and relative cell viability was determined to the scr control transfected cells. *P < 0.05; **P < 0.01; ns: not significant. Error bars represent the standard error of the mean (SEM) from three independent experiments. (E) Representative images of replication fork DNA fibers in both wild‐type and mutant KRAS lines. Fig. S3. (A) Representative cytogram images of cell cycle distribution analyzed at 6 h after exposure to IR (6 Gy) in the isogenic lines using ModFit LT 4.0 software. (B) Percentage of cell cycle distribution analyzed at 24 h following 1 Gy of IR treatment in the isogenic lines using ModFit LT 4.0 software. n = 2 ± SEM. (C) Representative images of the isogenic colorectal cancer lines coimmunostained with anti‐53BP1 (green), Cyclin A (red) and DAPI (blue) following 1 Gy ionizing radiation processed after 0.5 or 24 h. Fig. S4. (A) DKs‐8 cells were reverse transfected with either 10 nm of siRNA (left) or treated with 0.25 μm AZD6244, a MEK1/2 inhibitor and cell viability was determined after 96 h. Relative cell viability was determine [file MOL2-11-470-s001.pdf]

Extrapolating genetic interactions from *S.cerevisiae* identifies enhanced dependency of KRAS mutant colorectal cancer cells on RAD51-dependent homologous recombination repair

Murugan Kalimutho, Amanda L Bain, Bipasha Mukherjee, Purba Nag, Devathri M Nanayakkara, Sarah K Harten, Janelle L Harris, Goutham Narayanan Subramanian, Debottam Sinha, Senji Shirasawa, Sriganesh Srihari, Sandeep Burma and Kum Kum Khanna

Supplementary Figure 1

A.

```
KRAS    -------MTEYKLVVVGAGGVGKSALTIQLIQNHFVDEYDPTIEDSYRKQVVIDGETCLL   53
HRAS    -------MTEYKLVVVGAGGVGKSALTIQLIQNHFVDEYDPTIEDSYRKQVVIDGETCLL   53
NRAS    -------MTEYKLVVVGAGGVGKSALTIQLIQNHFVDEYDPTIEDSYRKQVVIDGETCLL   53
RAS1    MQGNKSTIREYKIVVVGGGGVGKSALTIQFIQSYFVDEYDPTIEDSYRKQVVIDDKVSIL   60
RAS2    MPLNKSNIREYKLVVVGGGGVGKSALTIQLTQSHFVDEYDPTIEDSYRKQVVIDDEVSIL   60
                :  ***:****.************:  *.:********************  :..:*

KRAS    DILDTAGQEEYSAMRDQYMRTGEGFLCVFAINNTKSFEDIHHYREQIKRVKDSEDVPMVL   113
HRAS    DILDTAGQEEYSAMRDQYMRTGEGFLCVFAINNTKSFEDIHQYREQIKRVKDSDDVPMVL   113
NRAS    DILDTAGQEEYSAMRDQYMRTGEGFLCVFAINNSKSFADINLYREQIKRVKDSDDVPMVL   113
RAS1    DILDTAGQEEYSAMREQYMRTGEGFLLVYSVTSRNSFDELLSYYQQIQRVKDSDYIPVVV   120
RAS2    DILDTAGQEEYSAMREQYMRNGEGFLLVYSITSKSSLDELMTYYQQILRVKDTDYVPIVV   120
        ***************:****.***** *::::..  .*: ::  * :** ****:: :*:*:

KRAS    VGNKCDLPSR-TVDTKQAQDLARSYGIPFIETSAKTRQGVDDAFYTLVREIRKHKEKMSK   172
HRAS    VGNKCDLAAR-TVESRQAQDLARSYGIPYIETSAKTRQGVEDAFYTLVREIRQHKLRKLN   172
NRAS    VGNKCDLPTR-TVDTKQAHELAKSYGIPFIETSAKTRQGVEDAFYTLVREIRQYRMKKLN   172
RAS1    VGNKLDLENERQVSYEDGLRLAKQLNAPFLETSAKQAINVDEAFYSLIRLVRDDGGKYNS   180
RAS2    VGNKSDLENEKQVSYQDGLNMAKQMNAPFLETSAKQAINVEEAFYTLARLVRDEGGKYNK   180
        **** **  .  *. .:.  :*:.   *::*****    *::***:* * :*.    :  .

KRAS    DGK-K---KK------KK----------SKTKCVIM------------------------   188
HRAS    PPDES---GP------GC----------MSCKCVLS------------------------   189
NRAS    SSDDG---TQ------GC----------MGLPCVVM------------------------   189
RAS1    MNRQLDNTNEIRDSELTSSATADREKKNN-GSYVLDNSLTNAGTGSS------------S   227
RAS2    TLTENDNSKQTSQDTKGSGA--NSVPRNSGGHRKMSNAANGKNVNSSTTVVNARNASIES   238
                                           :

KRAS    ------------------------------------------------------------   188
HRAS    ------------------------------------------------------------   189
NRAS    ------------------------------------------------------------   189
RAS1    KSAVNHNGETT--KRTDEKNYVNQNNNNEGNTKY-SSNGNGNR--SDISRGNQNNALNSR   282
RAS2    KTGLAGNQATNGKTQTDRTNI-DNSTGQAGQANAQSANTVNNRVNNNSKAGQVSNAKQ-A   296

KRAS    ---------------------------   188
HRAS    ---------------------------   189
NRAS    ---------------------------   189
RAS1    SKQSAEPQKNSSANARKESSGGCCIIC   309
RAS2    RKQQAAPGG-NTSEASKSGSGGCCIIS   322
```

B.

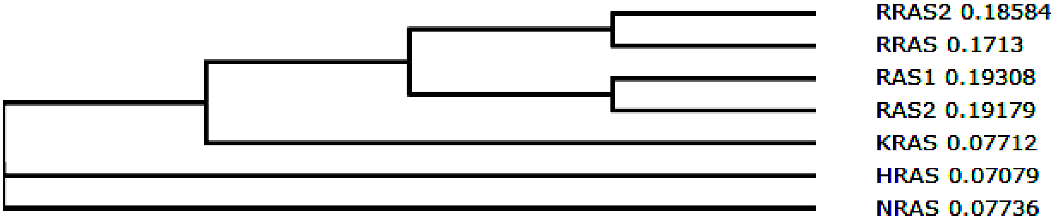

Supplementary Figure 2

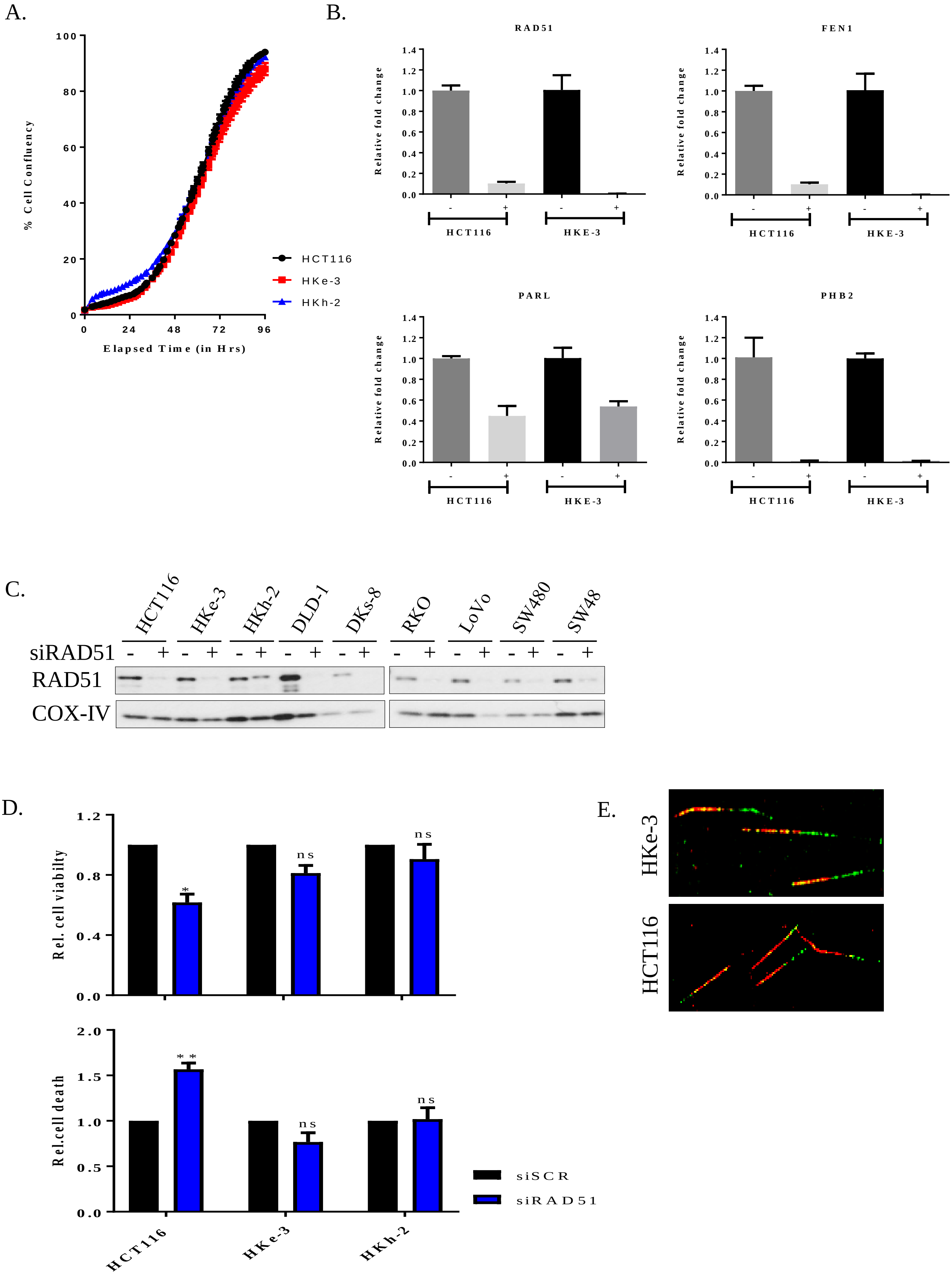

Supplementary Figure 3

A.

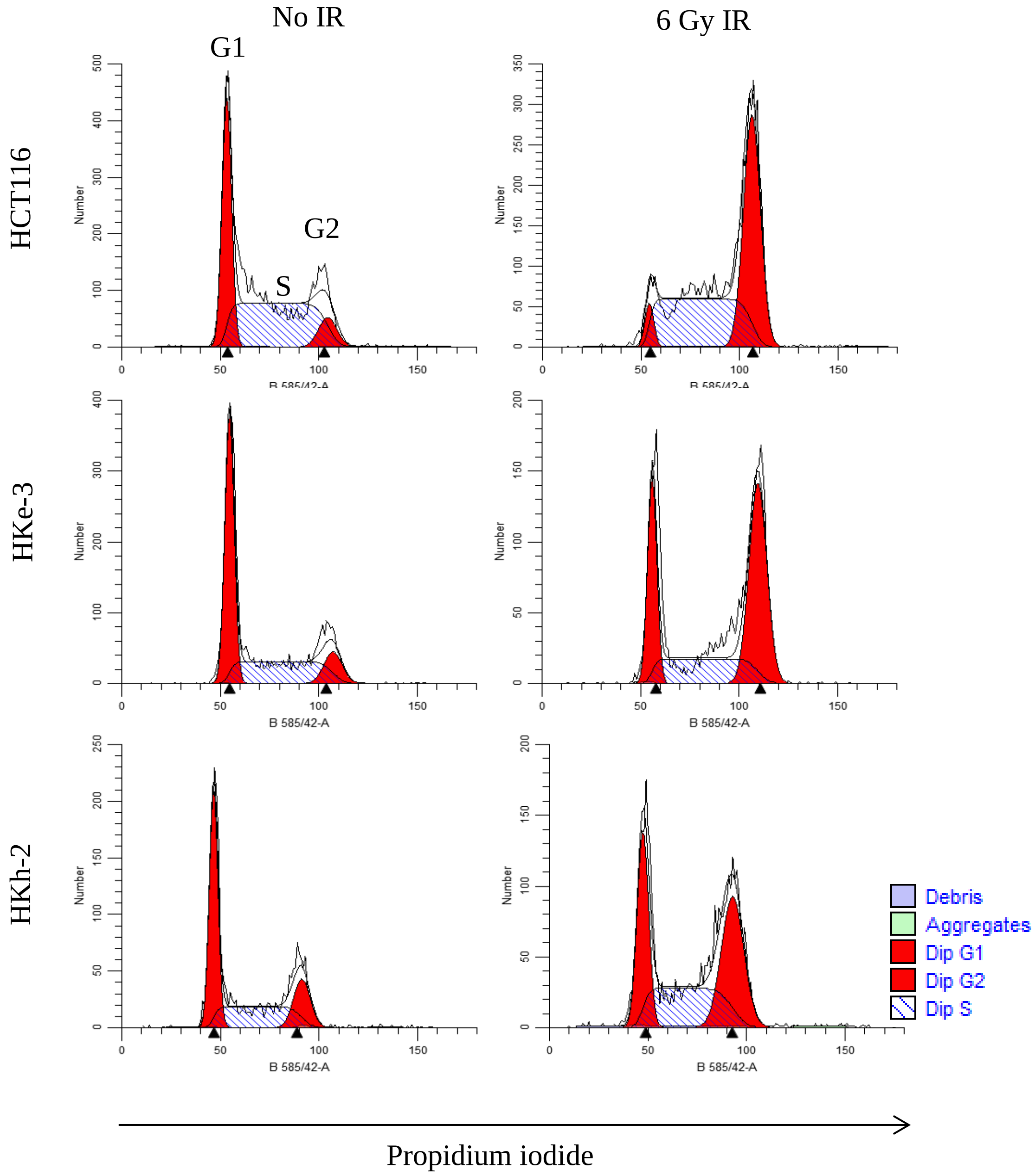

B.

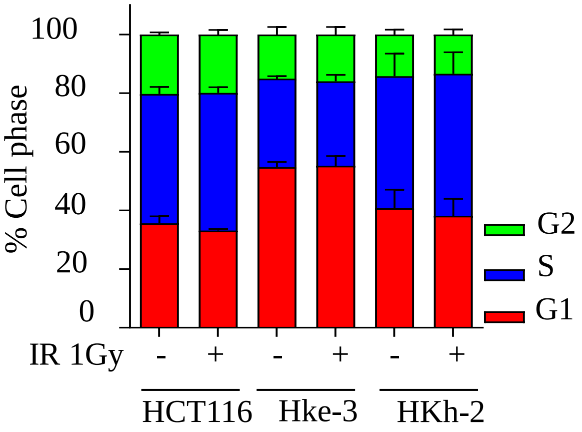

C.

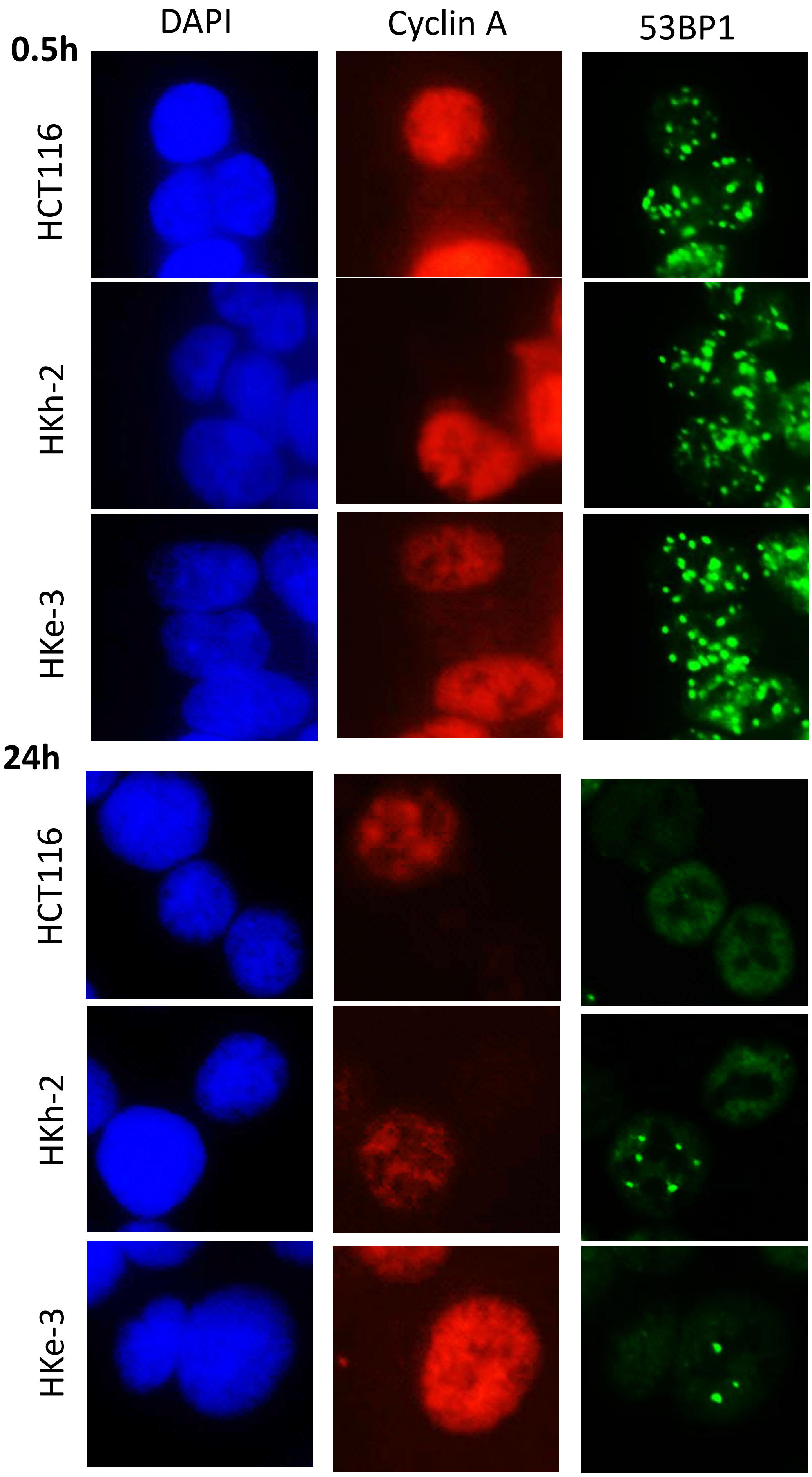
DAPI
Cyclin A
53BP1
0.5h
HCT116
HKh-2
HKe-3
24h
HCT116
HKh-2
HKe-3

Supplementary Figure 4

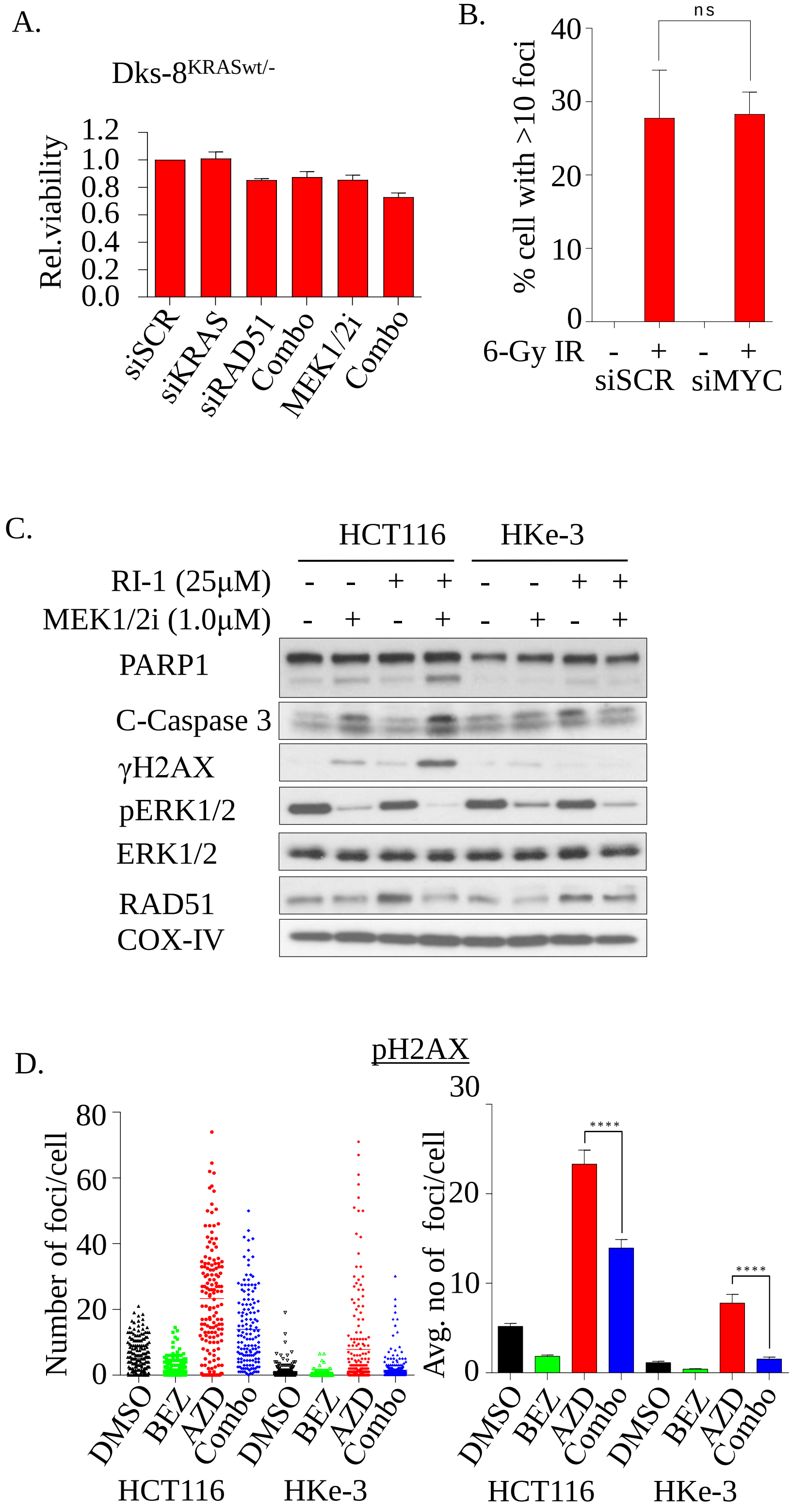

Supplementary table 1 : Primer sequences used in this study

Gene Name	Sequence	
	Forward（5'-3'）	Reverse（5'-3'）
RAD51	TCACGGTTAGAGCAGTGTGG	TTAGCTGCCTCAGCCAGAAT
FEN1	CCTGATGGGCATGTTCTACC	GCACTCATCATTGTGCTGCT
PARL	CAGCGGACTGTGACAGGTAT	AAATGACTGAATGTTGACAGCAA
PHB2	CCAAGGGATCCTGAAGTGAA	GCCCTGAAGCTGTTGCTG
ACTB	CCCAGAGCAAGAGAGAGG	GTCCAGACGCAGGATG

Supplementary table 2 : siRNA sequences used in this study

Gene Name	Sequence	
	sense（5'-3'）	antisense（5'-3'）
Hs_ELP2_3	GCCAGACAUUGUAGAGUUUCGCAAG	CUUGCGAAACUCUACAAUGUCUGGCAG
Hs_PARL_1	GGACAGUAGUGGUGCAUCUGGUCCT	AGGACCAGAUGCACCACUACUGUCCAA
Hs_PHB2_3	CCUCAUCAAGGGUAAGAAAUGAGCC	GGCUCAUUUCUUACCCUUGAUGAGGCU
Hs_DAD1_4	CAGCCUCUGCCUUUCAUUAAAUGT	UACAUUUAAUGAAAGGCAGAGGCUGGA
Hs_KRAS _2	CCUGCUCCAUGCAGACUGUUAGCTT	AAGCUAACAGUCUGCAUGGAGCAGGAA
Hs_KRAS_3	GGUGCAUGCAGUUGAUUACUUCUTA	UAAGAAGUAAUCAACUGCAUGCACCAA
Hs_RAD51_1	CCAUCUACCUGCUUGGUCUUUCATT	AAUGAAAGACCAAGCAGGUAGAUGGUG
Hs_RAD51_2	GUGCUGCAGCCUAAUGAGAGUGCAC	GUGCACUCUCAUUAGGCUGCAGCACUU
Hs_RAD51_3	GGAAGACCCAGAUCUGUCAUACGCT	AGCGUAUGACAGAUCUGGGUCUUCCCA
Hs_FEN1_1	GCAGCACAAUGAUGAGUGCAAACAT	AUGUUUGCACUCAUCAUUGUGCUGCUU
Hs_FEN1_2	CCACUUCUCAGGCAGUUUAAUGGAC	GUCCAUUAAACUGCCUGAGAAGUGGUU
Hs_FEN1_3	GCAGCACAAUGAUGAGUGCAAACAT	AUGUUUGCACUCAUCAUUGUGCUGCUU
Hs_MYC_5	AUGUAAACUGCCUCAAAUUGGACTT	AAGUCCAAUUUGAGGCAGUUUACAUUA
Hs_MYC_6	GCGACGAGGAGGAGAACUUCUACCA	UGGUAGAAGUUCUCCUCCUCGUCGCAG
Hs_ETS1_4	CCCAGAGAUGCCUUAACCUUUGUTG	CAACAAAGGUUAAGGCAUCUCUGGGAA
Hs_ETS1_1	CCAGAAGAGAGGAAUGACUUGAAGG	CCUUCAAGUCAUUCCUCUCUUCUGGAA
